# Toward a Balanced Description of Ground and Excited States with Transcorrelated F12 Methods

**DOI:** 10.1021/acs.jctc.5c01434

**Published:** 2025-10-13

**Authors:** Conner Masteran, Bimal Gaudel, Edward F. Valeev

**Affiliations:** Department of Chemistry, 1757 Virginia Tech, Blacksburg, Virginia 24061, United States

## Abstract

By correlating only the 1-particle states occupied in the reference determinant, the conventional design for the single-reference R12/F12 explicitly correlated methods biases them toward the ground-state description, thereby making the treatment of response properties of the ground state, and energies and other properties of excited states less robust. While the use of multireference methods and/or extensions of the standard SP-projected geminals can achieve a more balanced description of ground and excited states, here we show that the same goals can be achieved by extending the action of F12 correlators to the occupied and valence unoccupied 1-particle states only. This design choice reflects the strong dependence of the optimal correlation length scale of the F12 ansatz on the orbital energies/structure, and helps to avoid the unphysical raising of the ground-state energy if the F12 geminals are used to correlate pairs of all 1-particle states. The improved F12 geminal design is incorporated into the unitary transcorrelation framework to produce a unitary 2-body Hamiltonian that incorporates the short-range dynamical correlation physics for ground and low-energy excited states in a balanced manner. This explicitly correlated effective Hamiltonian reduces the basis set requirement on the correlation-consistent basis cardinal number by 1 or more over the uncorrelated counterpart for the description of ground-state coupled-cluster singles and doubles (CCSD) energies, vertical excitation energies, and harmonic vibrational frequencies of equation-of-motion CCSD low-energy excited states.

## Graphical abstract

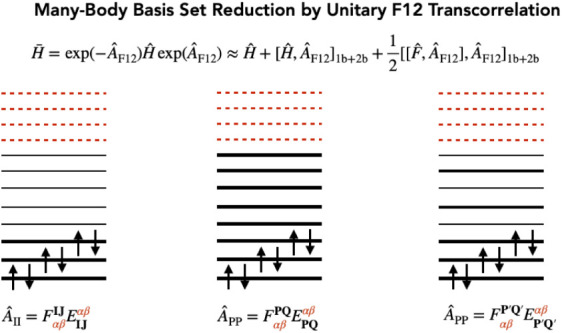

## 1 Introduction

The utility of interelectronic distances as explicit variables in the definition of many-electron wave functions has been known since the work of Hylleraas^1^ and Slater.^2,3^ The explicit use of such coordinates in the *explicitly correlated* methods greatly speeds up the basis set convergence by modeling more efficiently the known analytic behavior of the wave function near the electron–electron cusps of the exact wave function.^4,5^ There are several groups of explicitly correlated approaches in use today:

- **high-precision methods**: involve unfactorizable many-particle integrals with most or all particles at once that are evaluated analytically; examples include Hylleraas-CI,^6^ explicitly correlated Gaussian geminals,^7^ and free iterative complement interaction (ICI) approach of Nakatsuji.^8^
- **real-space Jastrow factor**: methods involving unfactorizable many-particle integrals with most or all particles at once that are evaluated stochastically or avoided by serving as a trial wave function; examples include single- and multiconfiguration Slater-Jastrow wave functions in variational and diffusion quantum Monte Carlo methods.^9^
- **real-space transcorrelation**: involves unfactorizable integrals with at most 3 particles that are evaluated analytically;^10,11^

- **R12/F12 methods**: involve unfactorizable integrals with up to 4 particles that are evaluated analytically (up to 2-particle integrals) or by the resolution-of-the-identity (3- and 4-particle integrals).^12–17^

By only requiring the exact evaluation of (nonstandard) 2-particle integrals the F12 methods are most practical among all explicitly correlated methods, available routinely in several packages and with demonstrated applications to hundreds and thousands of atoms.^18–21^ Another key advantage of modern F12 methods over most other explicitly correlated methods is that no problem-specific nonlinear optimization of the parameters of the explicitly correlated terms is required. This is due to the use of the explicitly correlated terms (“geminals”) to handle *only* the largely universal physics^4,5^ of the short-range electron correlation near the electron–electron cusp; the conventional superpositions of orbital products (Slater determinants) account for the rest of nonuniversal physics of longer-range correlations (such as dispersion interactions). Lastly, the spin dependence of the cusp conditions^5^ can be rigorously satisfied in the modern F12 framework^22^ unlike in the purely real-space methods.^23^

The vast majority of development and applications of the F12 framework have focused on the properties of ground states, such as thermochemistry and physics of noncovalent interactions, which are particularly impacted by the basis set errors from short-range electron correlations. Most of the time the lowest vertical excitation energies, on the other hand, often exhibit much smaller basis set errors. This is due to the robust cancellation of dynamical correlation errors in the ground and excited states. Nevertheless, the basis set errors can be substantial (especially for states with a Rydberg character^24^ and difficult to suppress. F12 methods are also expected to be important for an accurate description of excited state potential energy surfaces. Hence, it is highly desirable to deploy the F12 framework for the description of excited states and response properties. And, although multiple electronic states can be treated on equal footing by the F12 extensions of multiconfiguration wave function methods^24,25^ it is highly desirable to deploy the F12 framework in the context of conventional single-reference approaches like the coupled-cluster theory that can often model electron correlation of ground and low-energy excited states with unrivaled accuracy. A hindrance to such developments is that F12 methods recover more correlation energy for the ground than excited states leading to an imbalanced description between the states. This imbalance leads to artificially high vertical excitation energies, as was first observed by Fliegl et al.^26^ This imbalance in the description of ground vs excited states can be partially attributed to the construction of the geminal terms. The original orbital-invariant ansatz of the F12 theory^27^ and the de facto standard “SP” ansatz^22^ only explicitly correlate pairs of occupied (or *hole*, in the quasiparticle picture) orbitals. There have been several approaches that use geminals to also correlate particles in unoccupied (virtual, or *particle*) orbitals. Neiss, Hättig, and Klopper extended the set of geminal-generating orbitals to include a subset of particle orbitals selected according to the occupation numbers of MP2 densities.^28,29^ Köhn extended the SP ansatz to include geminal excitations from hole-particle orbital pairs.^30^ Köhn’s extended SP (XSP) ansatz has been used for description of response properties in the linear response coupled-cluster framework^30,31^ and for prediction of excitation energies in the context of Similarity Transformed Equation Of Motion Coupled Cluster (STEOM-CC) by Bokhan et al.^32^ Geminal-generating orbitals also have to include unoccupied orbitals in the context of energy-dependent F12 single-particle propagator methods.^33,34^

Although the use of geminals for correlating particles in the reference-unoccupied orbitals does improve the description of excitation energies and related quantities like response functions and self-energies, the price seems to be a *worse* description of the ground state compared to the SP ansatz.^30^ The XSP ansatz can address this issue by introducing additional parameters, such as in the XSP_opt_ approach,^30^ but unfortunately the numerical optimization of geminal-related amplitudes (practiced long before the introduction of the SP ansatz but largely abandoned since the SP ansatz introduction) can be ill-posed. In this paper we reexamine the problem of constructing the F12 ansatz appropriate for the low-lying excited states *without* sacrificing the accuracy of the SP ansatz for the ground states *and without* the need to introduce additional parameters (the latter is particularly important in the context of transcorrelated form of F12 methods used here). Our key hypothesis is that the single-parameter exponential correlation factors used in the modern F12 toolkit are tuned primarily for valence orbitals and are less appropriate for description of orbital pairs that include nonvalence orbitals. The idea that geminal definition must be adjusted for nonvalence orbitals is already well-known from studies of correlation energies including core electrons; e.g., Werner et al. found that the optimal correlation factor exponents differ substantially between core–core­(cc), core–valence­(cv), and valence–valence­(vv) orbital pairs.^35^ In general, the optimal exponents were the largest for cc contributions and the smallest for vv contributions.

Our investigation of the geminal basis construction will use the *a priori* form of the F12 technology, where the Hamiltonian is transformed using the F12 wave operator first. A particular form of F12 transcorrelation used here is based on the approximate unitary (canonical) transcorrelated approach of Yanai and Shiozaki^36^ who referred to the approach as CT-F12 and used by ourselves^37,38^ and others^39^ to significantly reduce the basis set errors. The errors in the original formalism and its implementation were recently corrected using an automated implementation.^40^ The unitary F12 TC formalism was shown to be as robust as traditional F12 approaches for ground states.^40^ Unlike the real-space transcorrelation of Boys and Handy^10,11,41,42^ which is the subject of intense recent attention,^43–47^ the F12-style transcorrelation avoids nonlinear optimization and spin-contamination. The unitary F12 transcorrelation also has some advantages over the nonunitary counterpart^48^ in that the transformed (downfolded) Hamiltonian is Hermitian and only includes 2-particle interactions, hence it can be used with most electronic structure models without modification. Here we used it in the context of excited-state single-reference coupled-cluster models.

The rest of the paper is structured as follows. In Section 2 we describe the unitary F12 TC formalism and introduce several new F12 geminal ansatze. Section 3 contains the pertinent technical details of our computational experiments. In Section 4 we report on the performance of the standard and new F12 geminal ansatze for ground- and excited-state energies and properties. Section 5 contains a summary of our findings.

## 2 Formalism

### 2.1 Hermitian F12 Transcorrelation

Transcorrelated Hamiltonians are generated by a similarity transformation with the operator *e* ^*Â*^, where *Â* is an operator that includes dependence on the interparticle distances and designed such that the TC Hamiltonian,

1 H̅^=e−ÂĤeÂ

is free of 2-electron singularities. In the original transcorrelation method of Boys and Handy^10,11,41^ and its subsequent reincarnations^43,49–51^ *Â* is a multiplicative configuration-space operator which can assume very general forms,^52^ with the resulting *non-Hermitian* TC Hamiltonian having a closed analytic form usually including up to 3-particle terms. In contrast, projective transcorrelation methods use a Fock-space correlator *Â* constructed in the spirit of R12/F12 methods; the resulting TC Hamiltonians do not have an analytic form but can be evaluated efficiently using the robust approximations of the R12/F12 methods. The original projective transcorrelation approach of Yanai–Shiozaki produced a *Hermitian* TC Hamiltonian as a nontruncating Baker–Campbell-Hausdorff (BCH) series by using an *antihermitian Â* and ad hoc truncating the BCH series to ensure cancellation of Coulomb electron–electron singularities to first order; it is not a coincidence that the resulting complexity of the many-particle integrals (and thus the short-range correlation physics) is similar to that of the MP2-F12 and approximate CCSD-F12 methods.^53–55^ Although the recently proposed *non-Hermitian* projective transcorrelation *Â* can formally ensure rigorous truncation of the BCH series for *H̅̂* at high particle ranks by using a general excitation-only Fock-space *Â*, in practice any projective transcorrelation method must truncate the BCH series to avoid a runaway growth of the particle rank of TC Hamiltonian. Thus, the Hermitian vs non-Hermitian design pivot is unlikely to grant a definitive advantage.

Thus, here we will focus on the Hermitian projective TC approach that was originally investigated^36,40^ with *Â* defined to introduce correlations only between pairs of *active* reference-occupied single-particle (*sp*) states {*i*} (see Figure 1 for the sp space glossary):

2 $${Â}_{\mathrm{II}}=\frac{1}{2}\left(G_{\alpha_{1}\alpha_{2}}^{i_{1}i_{2}}{Ê}_{i_{1}i_{2}}^{\alpha_{1}\alpha_{2}}-{G^{†}}_{i_{1}i_{2}}^{\alpha_{1}\alpha_{2}}{Ê}_{\alpha_{1}\alpha_{2}}^{i_{1}i_{2}}\right)\equiv \frac{1}{2}\left(G_{\alpha_{1}\alpha_{2}}^{i_{1}i_{2}}{Ê}_{i_{1}i_{2}}^{\alpha_{1}\alpha_{2}}-h.c.\right)$$

where *h.c.* denotes the Hermitian conjugate of the preceding terms. Here and elsewhere, the Einstein summation convention of duplicate indices is assumed, unless noted explicitly. In eq 2 *Ê* is the *spin-free* Fermionic 2-particle transition operator normal-ordered with respect to the genuine (0-particle) vacuum:

3 $${Ê}_{\kappa_{1}\kappa_{2}}^{\kappa_{3}\kappa_{4}}=\underset{\sigma \tau }{\sum }{â}_{\kappa_{3}\sigma }^{†}{â}_{\kappa_{4}\tau }^{†}{â}_{\kappa_{2}\tau }{â}_{\kappa_{1}\sigma }$$

with σ and τ enumerating the spin state basis, and *G* and *G* ^†^ are the matrix elements of the standard SP ansatz projected geminal:^22,56^

4 $$G_{\alpha_{1}\alpha_{2}}^{i_{1}i_{2}}=\left\langle \alpha_{1}\alpha_{2}\right|Ĉ{\mathrm{Q̂}}_{12}f\left(r_{12}\right)\left|i_{1}i_{2}\right\rangle$$

5 $${G^{†}}_{i_{1}i_{2}}^{\alpha_{1}\alpha_{2}}=\left\langle i_{1}i_{2}\right|f\left(r_{12}\right){\mathrm{Q̂}}_{12}Ĉ\left|\alpha_{1}\alpha_{2}\right\rangle$$

with *Ĉ* = (3 + *P̂*_12_)/8 adapting the correlator to the S- and P-wave cusp conditions^5^ (*P̂*_12_ is swaps particles 1 and 2). The projector *Q̂*_12_ used in this work,

6 $${\mathrm{Q̂}}_{12}=1-{\mathrm{V̂}}_{1}{\mathrm{V̂}}_{2}$$

corresponds to the standard ansatz 2 of the R12/F12 theory^57,58^ (some denote it as ansatz 3.^59^ whose effect is to exclude the conventional intra-OBS replacements from *Â* that are going to be accounted for by the conventional correlation treatment that follows the transcorrelation. That is, eq 6 excludes the contributions of double replacements from the active occupied orbitals to all unoccupied orbitals (that is, $E_{i_{1}i_{2}}^{e_{1}e_{2}}$ ). The standard single-parameter Slater-type geminal (STG),^22^ $f\left(r_{12}\right)=-\frac{e^{-\gamma r_{12}}}{\gamma }$ , is used, with the recommended value^60^ of the inverse length scale γ optimized a priori and tabulated for the most common OBS.

**Figure 1:**
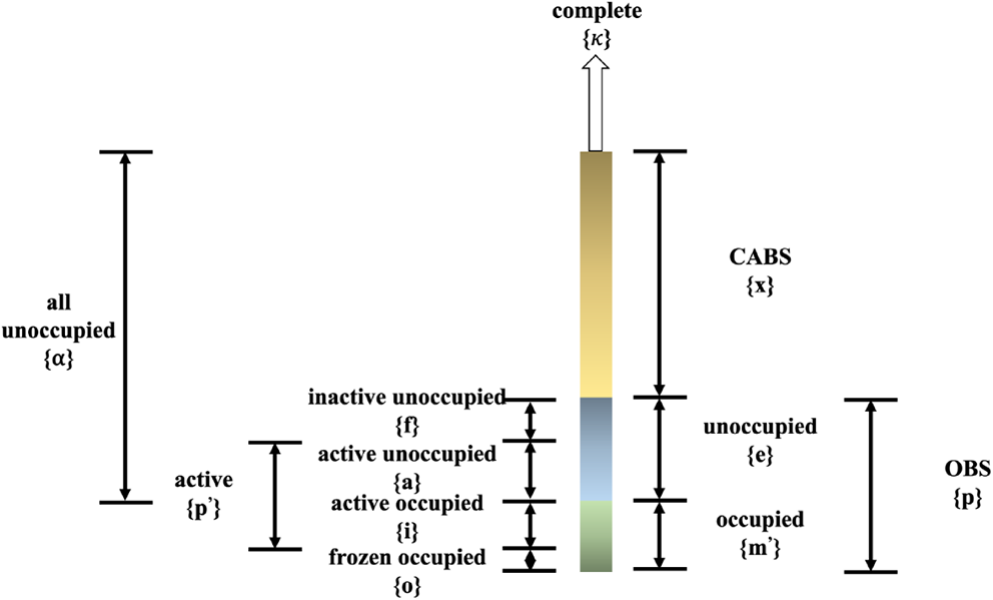
Schematic diagram of the single-particle state spaces used in this text.

With this in place we can define an effective singularity-free Hamiltonian by the correlator it contains:

7 H̅^II=e−ÂIIĤeÂII

Although the BCH expansion of eq 1 generated by the antihermitian *Â* does not truncate, the leading-order cancellation of singularities is ensured by keeping the up to quadratic terms:^36,40^

8 $$e^{-Â}Ĥe^{Â}\approx Ĥ+[Ĥ,Â{]}_{12}+\frac{1}{2}[\left[\mathrm{F̂},Â\right],Â{]}_{12}$$

where *F̂* is the Fock operator. The single and double commutator terms in eq 8 contain up to 3-particle operators that are further approximated (as denoted by “_12_”) by rewriting them in normal order with respect to a reference state and neglecting the resulting 3-particle normal-ordered operators and cumulants of the 3-particle reduced density matrix (RDM) of the reference.^61–63^

The resulting TC Hamiltonian is Hermitian and contains up to 2-particle terms. As documented in Ref. 40 the performance of transcorrelation for ground-state CC methods with up to quadruple excitations matches or beats the performance of the conventional CC-F12 methods, by reducing the basis set error of the correlation energy by an equivalent of 2 cardinal numbers.

Unfortunately, the TC Hamiltonian produced with eq 2 is not appropriate for the treatment of excited states due to its bias toward the ground state. There are several sources of the bias. The first (and most important) factor is the use of states that are occupied and unoccupied in the ground-state reference in the correlator (eq 2). The second factor is the use of the ground-state Fock operator in eq 8. The third factor is the use of a zeroth-order ground state reference for defining the cumulant-based screening of the 3-particle terms. And the last factor is the use of reference-unoccupied states in projector *Q̂*_12_ (eq 6).

The first step toward eliminating these sources of bias was to generalize *Â* to correlate pairs of electrons in any active state *p*′ rather than in any occupied states:

9 $${Â}_{\mathrm{PP}}=\frac{1}{2}\left(G_{\alpha_{1}\alpha_{2}}^{p_{1}^{\prime }p_{2}^{\prime }}{Ê}_{p_{1}^{\prime }p_{2}^{\prime }}^{\alpha_{1}\alpha_{2}}-h.c\right)$$

To distinguish correlators in eq 2 and eq 9 we will refer to them as II and PP, respectively. Note that both are sufficiently general to be usable in both single- and multideterminantal reference states. Although neither ansatz II nor PP ensure full state universality, we expect ansatz PP to provide a better description of low-lying excited states. Relative performance of ansatze II and PP was recently documented by Kumar et al.^38^ but the use of near-minimal bases did not allow us to generalize the findings. The goal of this manuscript is to rectify this gap.

A natural half-step from II to PP ansatz is the hybrid IP ansatz:

10 $${Â}_{\mathrm{IP}}=\left(G_{\alpha_{1}\alpha_{2}}^{i_{1}p_{1}^{\prime }}{Ê}_{i_{1}p_{1}^{\prime }}^{\alpha_{1}\alpha_{2}}-h.c\right)-{Â}_{\mathrm{II}}$$

where the subtraction of *Â*_II_ is used to avoid double inclusion of the II correlations. Such choice of *Â* introduces correlations between pairs of electrons in which at least one electron is in an occupied orbital *i* and the other is in any active orbital *p*′. Although this ansatz seems to be an artificial choice for a correlator, its strong connection to the F12 ansatz used^30,31^ in the context of linear response coupled-cluster F12 methods makes it a useful point of comparison.

In this paper we compare the performance of the three correlator ansatze for ground and excited state properties. The use of automated symbolic tensor algebra framework SeQuant, which was essential for correct implementation of the II version of the F12 TC Hamiltonian in Ref. 40 allows straigtforward implementation of the IP and PP generalizations. Although the IP ansatz equations are longer than their II and PP counterparts due to the more complex form of eq 10, all three ansatze can be implemented with the same 3 “special” F12 intermediates V, X, and B, which for the PP ansatz read:

11 $$V_{p_{1}^{\prime }p_{2}^{\prime }}^{p_{1}p_{2}}=g_{\alpha_{1}\alpha_{2}}^{p_{1}p_{2}}G_{p_{1}^{\prime }p_{2}^{\prime }}^{\alpha_{1}\alpha_{2}}$$

12 $$X_{p_{1}^{\prime }p_{2}^{\prime }}^{p_{3}^{\prime }p_{4}^{\prime }}=G_{\alpha_{1}\alpha_{2}}^{p_{3}^{\prime }p_{4}^{\prime }}G_{p_{1}^{\prime }p_{2}^{\prime }}^{\alpha_{1}\alpha_{2}}$$

13 $$B_{p_{1}^{\prime }p_{2}^{\prime }}^{p_{3}^{\prime }p_{4}^{\prime }}=G_{p_{1}^{\prime }p_{2}^{\prime }}^{\alpha_{1}\alpha_{2}}f_{\alpha_{1}}^{\alpha_{3}}G_{\alpha_{3}\alpha_{2}}^{p_{3}^{\prime }p_{4}^{\prime }}+G_{p_{1}^{\prime }p_{2}^{\prime }}^{\alpha_{1}\alpha_{2}}f_{\alpha_{2}}^{\alpha_{3}}G_{\alpha_{1}\alpha_{3}}^{p_{3}^{\prime }p_{4}^{\prime }}$$

Generalization of eqs 11–13 to the IP case is straightforward. Intermediates V and X are evaluated using the CABS+ approach^58^ and intermediate B is evaluated using the so-called approximation C.^64^

### 2.2 Valence-Constrained Geminals

Unfortunately, the use of IP and PP ansatze produces ground state energies that are consistently higher than those from the more restrictive II ansatz. There is some precedent in the literature for such raising of the ground state correlation energy by straightforward use of geminals for correlation of virtual orbitals. Namely, Figures 2 and 3 in Andreas Köhn’s paper^30^ show that *extended* SP (XSP) F12 ansatz correlating not only pairs of occupied orbitals but also the occupied-virtual pairs similarly decreases the magnitude of the ground state correlation energy. The decrease in magnitude due to the use of XSP on the order of 0.1 mE_h_ for the aug-cc-pVTZ valence CCSD energy of the BH molecule, which is smaller than the ∼0.5 mE_h_ raising of the energy by our method with the IP correlator. Perhaps the scale of this issue is somehow magnified by the projective transcorrelated Hamiltonian approach, however, it is difficult to say, as ground-state energies are often left unreported in studies targeting excited-state properties. Nevertheless, this is an issue that we encountered and looked to address.

**Figure 2:**
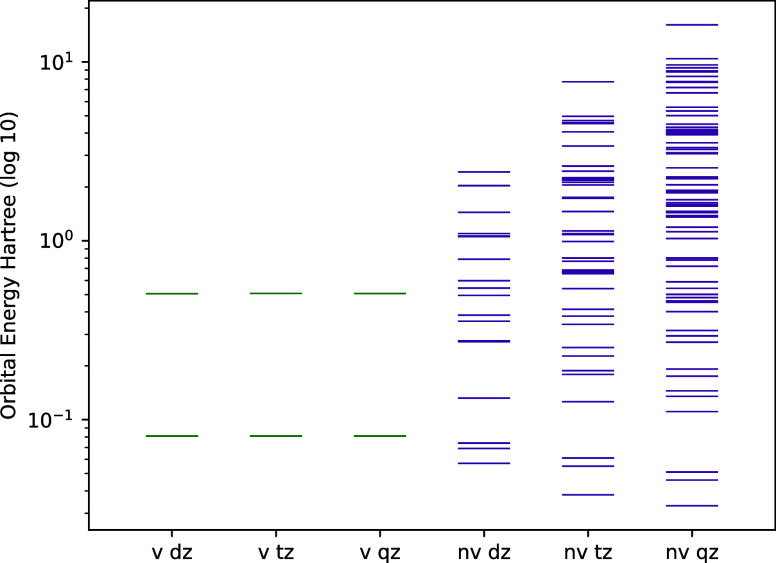
Pseudoeigenvalues of the unoccupied orbitals of BH in aug-cc-pVXZ basis sets. The valence orbitals (v) are shown on the left in green while the remaining nonvalence (nv) unoccupied orbitals are seen on the right in purple. Notice that the size of the active space is independent of the chosen orbital basis set.

Our experimentation hinted that the F12 geminals with globally fixed geminal length-scale are suitable for describing correlations of orbitals of the corresponding energy scales. This phenomenon is already well-known from the F12 studies of core correlation; the optimal geminal length-scales for correlation of core orbitals are shorter. Thus, it makes sense that the optimal length-scale for correlation of high-energy virtual orbitals will be substantially different from that of valence orbitals.

Therefore, we postulated that the optimal use of F12 correlators is to tune them for valence orbitals only. To test this hypothesis, we implemented variants of IP and PP correlators that use a valence subset of virtual orbitals only in their geminal-generating spaces. To distinguish the valence orbitals correlated by the F12 terms from the rest, we refer to them as *active* virtuals (not to be confused with the use of term “active” in the multiconfiguration context).

The most natural definition of valence virtual orbitals (VVOs) is by projection onto the exact orbitals of ground-state atoms, which for simplicity is emulated by a finite minimal basis set (MBS) of AOs. Such definition was originally used by Lu et al.^65^ and later reformulated in terms of SVD by Schmidt et al.^66^ Note that such definition of VVOs is remotely related to the much earlier work of Iwata^67^ and is equivalent (modulo the direct use of SVD vs an equivalent use of eigendecomposition) to the much more recent AVAS formalism of Sayfutyarova et al.^68^ We describe the construction of VVOs here for completeness. Nonorthogonal MBS AOs {|ϵ⟩} are symmetrically orthonormalized a la Löwdin to produce orthonormal MBS AOs {|ϵ̅⟩}:

14 $$\left|{\mathrm{\epsilon ̅}}_{1}\right\rangle ={(s^{-1/2})}_{\epsilon_{2}}^{\epsilon_{1}}\left|\epsilon_{2}\right\rangle$$

where **s** is the MBS AO metric (overlap matrix). The overlap matrix of the orthonormal MBS AOs with the unoccupied orbitals {|*e*⟩} that are expanded in OBS AOs {|μ⟩},

15 $$\left|e\right\rangle =C_{\mu }^{e}\left|\mu \right\rangle$$

is obtained straightforwardfly from the overlap of OBS and MBS AOs:

16 $$\left\langle \mathrm{\epsilon ̅}|e\right\rangle ={(s^{-1/2})}_{\epsilon }^{\epsilon_{1}}\left\langle \epsilon_{1}|\mu \right\rangle C_{\mu }^{e}$$

The SVD decomposition of this overlap matrix produces singular values between 1 and 0, with the largest values being very close to 1. The corresponding right singular vectors **V** ^†^ (with the rows corresponding to the ket indices *e*) can be interpreted as the linear combinations of the OBS unoccupied orbitals that have the largest overlap with MBS; these define the valence/active unoccupied orbitals {|*a*⟩}:

17 $$\left|a\right\rangle ={(V^{†})}_{e}^{a}\left|e\right\rangle$$

At physically relevant geometries, the number of such orbitals *n*_*a*_ equals the rank of MBS minus the number of occupied orbitals. The remaining columns of **V** ^†^ define the inactive unoccupied orbitals {|*f*⟩}.

Both shapes and energies of VVOs are known to have a well-defined basis set limit, with weak dependence on the basis set as illustrated in Figure 2.

Whether or not VVOs are used only changes the definition of the active orbital space *p*′ (see Figure 1), which is used in the definition of PP and IP correlators (eqs 9 and 10, respectively). If VVOs are used, space *p*′ is the union of active occupied (*i*) and active/valence unoccupied (*a*) spaces. If VVOs are not used, then *p*′ is the union of active occupied space *i* and the full OBS unoccupied space *e*. Whether or not the geminals are constrained to the valence subspace of virtuals, the expressions for the IP and PP correlators remain unchanged. The projector eq 6 is also unchanged as it includes projection onto the full set of OBS unoccupied orbitals, which preserves the standard expressions for F12 intermediates.

## 3 Technical Details

The existing implementation of the transcorrelated F12 Hamiltonian with the II correlator^40^ in the developmental version of the MPQC package^69^ was extended to support the use of IP and PP correlators, with optional restriction of the geminals to the VVO subset of virtuals.

The transcorrelated Hamiltonians are derived using the symbolic tensor algebra package SeQuant. The resulting tensor algebraic expressions are optimized (“factorized”) and evaluated numerically. The F12 Hamiltonians can then be used as the starting point for a correlated calculation or saved to disk for offline use. The use of IP and PP correlators leads to the appearance of standard intermediates V, X, and B of the F12 methods with geminal ansatz 2 (eqs 11–13) whose robust evaluation using approximate resolution of the identity is well-established.^58,64^ Whereas the original implementation of the TC F12 Hamiltonian with the II ansatz^40^ used the existing manually produced implementation of these intermediates, here the programmable expressions for these intermediates for the II, IP, and PP cases were generated by SeQuant, which was crucial for extensive experimentation with alternative ansatze that led to this work. The relevant details are discussed further in the Supporting Information.

Ten-no’s Slater-type geminal $\left(-\frac{e^{-\gamma r_{12}}}{\gamma }\right)$ was used as the correlation factor.^70^ AO integrals involving the geminal were computed directly, without the usual approximation by a linear combination of Gaussian Geminals.^71–73^

The standard aug-cc-pV*X*Z (shortened to a*X*Z),^74,75^ their doubly augmented daug-cc-pV*X*Z variants (shortened to da*X*Z),^76^ and the cc-pV*X*Z-F12 basis sets (shortened to *X*Z-F12)^60^ were used as orbital basis sets (OBS), with the recommended values of geminal exponents used (γ = {1.1, 1.2}*a*_0_^–1^ for *a*{D,T}­Z OBS, γ = {0.9, 1.0}*a*_0_^–1^ for {D,T}­Z-F12 OBS, and γ = {1.1, 1.3, 1.5}*a*_0_^–1^ for da­{D,T,Q}­Z OBS). The matching OptRI basis sets of Peterson and coworkers^77^ used to define the CABS space (using the CABS+ approach^57^ and to approximate the F12 intermediates. All 2-electron integrals were approximated by density fitting in aug-cc-PV­(*X* + 1)­Z-RIFIT^78^ for the corresponding OBS with cardinal number *X*. All VVOs are constructed from the MINI MBS.^79,80^

The II, IP, and PP correlators used to generate TC Hamiltonians did not correlate core electrons (frozen core approximation). The ground states of the TC F12 Hamiltonian were obtained by complete reoptimization of the Hartree–Fock orbitals with the F12 Hamiltonian (which produces the energy already including some dynamical correlation effects) and the subsequent coupled-cluster singles and doubles method. The excited states of the TC F12 Hamiltonian were obtained using the frozen-core EOM-CCSD (aka SAC–CI) method^81,82^ whose implementation was previously described.^83^

Equilibrium bond distances and harmonic vibrational frequencies of excited states were obtained by fitting the potential energy surfaces (PES) evaluated on an equidistant grid with spacing 10^–3^ *a*_0_ to a sixth-order polynomial, using the equilibrium geometries from ref.^84^ as the guide. Harmonic vibrational frequencies were reported for the most common isotopologue of each species.

## 4 Results

### 4.1 Vertical Excitation Energies

Tables 1–5 report the correlation contributions to the electronic energies of ground and excited states as well as vertical excitation energies obtained with standard and F12 TC Hamiltonians for several low-lying singlet states of 5 small molecules: BH, CH_2_O, N_2_, H_2_O, CH_2_. The BH geometry was taken from Kohn’s XSP paper,^30^ the CH_2_O geometry was taken from the benchmark study by Schreiber et al.,^85^ and the remaining three geometries were taken from the HEAT data set.^86^

**Table 1:** Comparison of Ground State and Lowest Lying Excited Singlet State Correlation Energies between the Various Ansatze for the BH Molecule^a^

		FVO			VVO		
Hamiltonian	Basis	$E_{c}^{0}$	$E_{c}^{1}$	Δ*E*	$E_{c}^{0}$	$E_{c}^{1}$	Δ*E*
*Ĥ*	aDZ	–89.862	–85.465	2.972			
	aTZ	–99.214	–96.741	2.930			
	aQZ	–101.766	–99.808	2.920			
*H̅̂*_II_	aDZ	–101.219	–93.492	3.062			
	aTZ	–102.757	–99.881	2.941			
*H̅̂*_IP_	aDZ	–99.812	–95.072	2.981	–100.416	–96.301	2.964
	aTZ	–102.161	–99.491	2.935	–102.444	–100.404	2.918
*H̅̂*_PP_	aDZ	–99.867	–95.194	2.979	–100.902	–96.825	2.963
	aTZ	–102.330	–99.534	2.939	–102.719	–100.465	2.924

a Correlation energies are reported in millihartree (*mE*_h_), vertical excitation energies are reported in electron volts (eV).

**Table 2:** Comparing Ground State Correlation Energy and Vertical Excitation Energies Generated by Various Ansatze for a Formaldehyde Molecule^a^

		FVO					VVO				
Hamiltonian	Basis	$E_{c}^{0}$	S_1_	S_2_	S_3_	S_4_	$E_{c}^{0}$	S_1_	S_2_	S_3_	S_4_
*Ĥ*	aDZ	–349.125	3.943	7.053	7.989	8.068					
	aTZ	–413.104	3.935	7.239	8.114	8.225					
	aQZ	–433.832	3.946	7.304	8.148	8.281					
*H̅̂*_II_	aDZ	–437.714	4.312	7.292	8.237	8.307					
	aTZ	–443.980	4.048	7.305	8.184	8.291					
*H̅̂*_IP_	aDZ	–438.125	4.010	7.272	8.208	8.295	–438.058	3.985	7.285	8.251	8.303
	aTZ	–443.384	4.001	7.311	8.189	8.296	–443.998	3.948	7.311	8.196	8.295
*H̅̂*_PP_	aDZ	–436.516	4.013	7.266	8.204	8.288	–438.487	4.006	7.287	8.256	8.307
	aTZ	–442.941	3.971	7.312	8.190	8.299	–444.366	3.960	7.313	8.200	8.298

a Correlation energies are reported in millihartree (*mE*_h_), vertical excitation energies are reported in electron volts (eV).

**Table 3:** N_2_ Singlet Ground State and First Few Excited Singlet States^a^

		FVO				VVO			
Hamiltonian	Basis	$E_{c}^{0}$	S_1_	S_2_	S_3_	$E_{c}^{0}$	S_1_	S_2_	S_3_
*Ĥ*	aDZ	–319.811	9.537	10.258	10.666				
	aTZ	–376.877	9.451	10.057	10.503				
	aQZ	–395.236	9.442	10.042	10.482				
*H̅̂*_II_	aDZ	–399.309	9.925	10.699	10.988				
	aTZ	–404.694	9.567	10.203	10.592				
*H̅̂*_IP_	aDZ	–399.646	9.589	10.297	10.698	–399.690	9.553	10.265	10.659
	aTZ	–404.265	9.480	10.089	10.524	–404.661	9.451	10.063	10.492
*H̅̂*_PP_	aDZ	–398.268	9.577	10.313	10.702	–400.112	9.560	10.307	10.681
	aTZ	–403.785	9.483	10.098	10.528	–405.066	9.464	10.090	10.508

a Correlation energies are reported in millihartree (*mE*_h_), vertical excitation energies are reported in electron volts (eV).

**Table 4:** CH_2_ Singlet Ground State and First Few Excited Singlet States^a^

		FVO				VVO			
Hamiltonian	Basis	S_0_	S_1_	S_2_	S_3_	S_0_	S_1_	S_2_	S_3_
*Ĥ*	DZ-F12	–154.014	1.718	5.757	6.139				
	TZ-F12	–167.784	1.679	5.716	6.257				
	QZ-F12	–172.195	1.673	5.716	6.292				
*H̅̂*_II_	DZ-F12	–172.867	1.859	5.923	6.334				
	TZ-F12	–175.401	1.718	5.772	6.312				
*H̅̂*_IP_	DZ-F12	–172.061	1.753	5.802	6.169	–172.487	1.735	5.785	6.182
	TZ-F12	–174.932	1.696	5.743	6.253	–175.209	1.681	5.730	6.258
*H̅̂*_PP_	DZ-F12	–171.768	1.765	5.814	6.090	–172.932	1.755	5.808	6.118
	TZ-F12	–174.873	1.702	5.747	6.225	–175.451	1.692	5.740	6.236

a Correlation energies are reported in millihartree (*mE*_h_), vertical excitation energies are reported in electron volts (eV).

**Table 5:** H_2_O Singlet Ground State and First Few Excited Singlet States^a^

		FVO				VVO			
Hamiltonian	Basis	S_0_	S_1_	S_2_	S_3_	S_0_	S_1_	S_2_	S_3_
*Ĥ*	aDZ	–230.040	7.082	8.847	8.923				
	aTZ	–275.393	7.215	8.985	9.018				
	aQZ	–290.443	7.277	9.045	9.070				
*H̅̂*_II_	aDZ	–293.187	7.341	9.104	9.148				
	aTZ	–297.276	7.293	9.062	9.083				
*H̅̂*_IP_	aDZ	–293.310	7.287	9.064	9.101	–293.086	7.299	9.072	9.116
	aTZ	–296.955	7.293	9.064	9.084	–297.185	7.292	9.060	9.085
*H̅̂*_PP_	aDZ	–292.421	7.285	9.059	9.098	–293.328	7.307	9.081	9.119
	aTZ	–296.489	7.293	9.063	9.083	–297.331	7.298	9.065	9.088

a Correlation energies are reported in millihartree (*mE*_h_), vertical excitation energies are reported in electron volts (eV).

Since the F12 geminals only account for the “2-particle” basis set incompleteness, conventional F12 approaches require perturbative account for the basis set incompleteness of the ground-state reference using single replacements into the CABS basis^54,87^ (such correction was already introduced in the context of dual basis MP2 method^88^). Whereas such a correction has been explored in the context of F12 TC framework before,^38^ our attempts to introduce such a correction in a state-universal manner were not successful, hence here we only focus on the improvements of the dynamical correlation energies that stem from the F12 transcorrelation. An alternative solution to the 1-particle basis set incompleteness problem will be reported elsewhere.

To gain deeper insight into the quality of vertical excitation energies obtained with the F12 transcorrelation Hamiltonians, it is useful to define quantitatively the correlation energies of not only the ground but also the excited states. For the ground states, we use the conventional (Löwdin) correlation energy:

18 Ec0≡⟨Ψ̅0|H̅^|Ψ̅0⟩−⟨Φ0|Ĥ|Φ0⟩

where |Φ^0^⟩ is the reference wave function. In this work ⟨Ψ̅^0^| and |Ψ̅^0^⟩ are the left- and right-hand CCSD ground-state wave functions obtained with the F12 TC Hamiltonian. For well-behaved ground states with the single-configuration character eq 18 includes primarily the dynamical correlation effects.

No unique excited-state counterpart to eq 18 is available. Here we defined the correlation energy of *s*th excited state as

19 Ecs≡⟨Ψ̅s|H̅^|Ψ̅s⟩−⟨Φs|Ĥ|Φs⟩

where |Φ^s^⟩ is the configuration interaction singles (CIS) excited-state wave function and ⟨Ψ̅^s^| and |Ψ̅^s^⟩ are the left- and right-hand EOM-CCSD wave functions of the *s*th excited state. eq 19 is expected to correspond to the dynamical component of the correlation energy of the excited state only if the CIS and EOM-CCSD states are qualitatively the same. Due to this limitation we only reported the excited state correlation energies for the lowest excited state of BH for which CIS and EOM-CCSD descriptions are qualitatively similar and this quantity provided useful insights into the successes and failures of the F12 TC approach.

Our analysis starts with the ground and lowest singlet excited state of BH (see Table 1), which were examined in detail with conventional excited-state CC-F12 approaches by Köhn.^30^ First, consider the data obtained with the full set of virtual orbitals (FVO). The II correlator (which most resembles the F12 cluster operators utilized in the CC-F12 methods aimed at ground states^53–55,89,90^ ) lowers the correlation energy by the equivalent of 2 cardinal quantum numbers. The II correlator also lowers the correlation energy of the excited state, but by a much smaller amount (the aDZ F12 energy is smaller in magnitude than the conventional aTZ counterpart). This is the primary reason why the net effect of the II correlator is to increase the vertical excitation energy, by almost 0.1 eV, rather than decrease it as expected from the basis set convergence of the standard excitation energy. The failure of the II correlator is due to the inability to correlate the single electron that was promoted from the occupied to the virtual orbital with the rest of the electrons.

We expected that the use of IP and PP correlators would resolve the undercorrelation of the excited state. Indeed, they do partially improve the quality of the excitation energy. However, replacing the II correlator by the more flexible IP or PP correlators unexpectedly *decreases* the magnitude of the ground state correlation energy, by about 1.5 and 0.5 mE_h_ with double and triple-ζ bases, respectively. As we discussed in Section 2, Köhn found similar issues in the context of exploring single-reference F12 coupled-cluster methods for excited states using his XSP (albeit on a smaller scale 0.1 mE_h_), however, this fact is not widely known and we were puzzled by such findings. In fact, the II correlator recovers more excited-state correlation energy at the aTZ level than both the IP and PP correlators using FVOs. When VVOs are used instead, the IP correlator recovers more ground-state correlation energy. This approach also produces improved excitation energies compared to its FVO counterpart. Similar improvement from the use of VVOs is observed for the PP correlator. The VVO PP correlator seems to strike the best balance between ground and excited-state correlation: it recovers almost as much ground state correlation as the II correlator while recovering substantially more correlation energy than the II correlator for the excited state.

All correlators significantly lower the ground state correlation energies of the formaldehyde molecule.Table 2. The FVO vs VVO contrast is especially striking here: in most instances the FVO-based correlators produce smaller correlation energy than the VVO counterpart. And again, the PP VVO correlator produces the lowest correlation energies with both aDZ and aTZ bases.

Like the case of BH where the lowest excited state was valence in character and therefore the excitation energy exhibited little basis set variation, the lowest excited state of formaldehyde has a valence character also, with nearly nonexistent dependence on the basis. As expected, the II correlator significantly overestimates this state’s excitation energy by 0.35 eV at the aDZ level by undercorrelating the excited electron. The use of IP and PP correlators addresses this issue nicely, with the VVO variants again superior to the FVO counterparts. Unlike the lowest excited state, the higher-lying S states of formaldehyde have a substantial Rydberg character thereby exhibiting a more protracted basis set convergence, with double-ζ excitation energies in error by 0.2–0.3 eV and converging from below. The use of VVO IP and PP correlators significantly reduces the basis set errors of the excitation energies for these 3 states, especially with the double-ζ basis. The S_3_ state is the only instance where the DZ and TZ F12 excitation energies differ by more than a couple of dozens of meV; this may suggest substantial 1-particle basis set incompleteness effects on the excitation energy of this state that the F12 transcorrelation does not incorporate in its present form.

Our findings are similar for the N_2_ molecule (Table 3) in that the VVO IP and PP correlators provide the best overall accuracy for the ground and excited states. The II correlator overestimates the excitation energies by as much as 0.5 eV, with the VVO IP and PP correlators reducing the error. However, unlike the case of formaldehyde, where the excitation energies of Rydberg-character states converged from below, here the excitation energies of all states converge from above. This again suggests the interplay between the 1- and 2-particle basis set incompleteness effects; this hypothesis is supported by the fact that the F12 transcorrelation (with the VVO IP and PP correlators) seems to have a relatively minor effect on the excitation energies; it is likely that the residual 1-particle basis set effects are important.

Only minor differences with the previous cases were observed for methylene (Table 4). The cc-pVXZ-F12 basis sets^60^ were used for this system due to issues converging TC-HF energies when using aug-cc-pVXZ basis sets. The use of cc-pVXZ-F12 basis sets purpose-built for F12 methods does not change the rate of convergence or magnitude of F12-spurred improvements: the ground-state energy again is improved by the equivalent of more than 2 cardinal basis set numbers. Out of the 3 excited states, excitation energies of two (S_1_ and S_2_) converge from above (like in N_2_) and one converges from below (like in CH_2_O). Thus, as expected, F12 transcorrelation (with VVO IP and PP correlators) does not seem to produce substantial effects on the excitation energies of the former and, somewhat unexpectedly, does not show substantial improvements for the latter. The interplay of the 1- and 2-particle basis set incompleteness effects is likely the cause of these differences.

Finally, the water molecule (Table 5) exhibits patterns similar to those of the other systems. With the excitation energies converging from below, the F12 transcorrelation greatly helps with the basis set convergence. The VVO PP correlator again provides the most balanced performance.

### 4.2 Excited State Properties

Unlike the excitation energies, which are often relatively weakly dependent on the basis set due to the frequently excellent cancellation of the basis set incompleteness errors between low-lying states, properties of individual excited states are just as sensitive to the basis set effects as their ground state counterparts. To probe the ability of the F12 TC approaches to reduce the basis set errors of excited state properties, we examined equilibrium geometries (Table 6) and harmonic vibrational frequencies (Table 7) of several paradigmatic diatomics (N_2_, CO, BF, and BH) obtained with standard explicitly correlated variants of EOM-CCSD energies. The F12 transcorrelated EOM-CCSD results obtained with the II and VVO-only form of PP correlators were compared against the standard EOM-CCSD and the conventional F12 form of the EOM-CCSD due to Yang and Hättig in which F12 terms are incorporated into the ground state clusters and the excited state wave vectors.^84^ Note that the EOM-CCSD­(F12) approach of Yang and Hättig uses the older orbital-invariant ansatz of the F12 theory^27^ in which each pair of orbitals is correlated by an adjustable superposition of geminals, rather than a single fixed-parameter geminal^22^ typically used in modern ground-state F12 methods as well as in the F12 transcorrelated approaches described here. The results are reported in Tables 6 and 7, respectively.

**Table 6:** Excited State Equilibrium Bond Lengths (pm) for Lowest Singlet Excited States of Several Small Diatomics^a^

		standard^b^					F12(II)		F12(VVO PP)		(F12)^c^	
	basis	daDZ	daTZ	daQZ	da5Z	CBS	daTZ	daQZ	daTZ	daQZ	daTZ	daQZ
N_2_	*a*′ Σu−1	126.7	125.4	125.1	125	124.9	125.1	124.9	125.1	124.9	125.1	124.9
	*a* ^1^Π_g_	122	120.7	120.3	120.2	120.1	120.4	120.2	120.4	120.1	120.4	120.2
	*w* ^1^Δ_u_	126.2	124.9	124.5	124.4	124.4	124.6	124.4	124.6	124.4	124.6	124.4
CO	*A* ^1^Π	124.5	123.1	122.5	122.4	122.3	122.7	122.3	122.7	122.4	122.6	122.3
	*B* ^1^Σ^+^	112.7	111.6	111.2	111.1	111	111.3	111	111.3	111	111.2	111
	*C* ^1^Σ^+^	112.6	111.5	111	110.9	110.8	111.1	110.9	111.1	110.9	111.1	110.9
BF	*A* ^1^Π	135.1	131.1	130.5	130.3	130.2	130.7	130.3	130.7	130.3	130.6	130.3
	*B* ^1^Σ^+^	123.7	121.2	120.7	120.6	120.5	120.9	120.6	121	120.6	120.9	120.6
	*C* ^1^Σ^+^	125.2	122.5	122	121.9	121.8	122.2	121.9	122.2	121.9	122.2	121.9
BH	*A* ^1^Π	124.4	122.4	122.2	122.1	122.1	122.2	122.1	122.2	122.1	122.3	122.1
	*B* ^1^Σ^+^	123.4	121.8	121.6	121.6	121.5	121.7	121.6	121.7	121.6	121.7	121.6

a F12-transcorrelated EOM CCSD approaches with II and VVO PP correlators are contrasted with the standard and conventional F12-correlated EOM CCSD formalisms.

b EOM-CCSD­(F12) results from ref. 84.

c Standard EOM-CCSD results from ref. 84.

**Table 7:** Harmonic Vibrational Frequencies (cm^–1^) for the Lowest Singlet Excited States of Several Small Diatomics^a^

standard^b^							F12(II)		F12(VVO PP)		(F12)^c^	
	basis	daDZ	daTZ	daQZ	da5Z	CBS	daTZ	daQZ	daTZ	daQZ	daTZ	daQZ
N_2_	*a*′ Σu−1	1709	1714	1725	1727	1730	1722	1727	1723	1728	1724	1729
	*a* ^1^Π_g_	1827	1830	1848	1851	1854	1846	1854	1846	1853	1847	1854
	*w* ^1^Δ_u_	1726	1733	1744	1746	1749	1742	1748	1744	1748	1744	1748
CO	*A* ^1^Π	1510	1561	1581	1587	1592	1574	1586	1574	1587	1580	1589
	*B* ^1^Σ^+^	2184	2221	2244	2249	2253	2241	2250	2241	2249	2246	2253
	*C* ^1^Σ^+^	2219	2259	2281	2285	2289	2277	2287	2276	2286	2282	2289
BF	*A* ^1^Π	1127	1258	1271	1275	1279	1267	1274	1272	1276	1275	1278
	*B* ^1^Σ^+^	1580	1704	1715	1718	1720	1715	1719	1712	1718	1717	1720
	*C* ^1^Σ^+^	1487	1620	1632	1635	1637	1632	1636	1639	1635	1633	1637
BH	*A* ^1^Π	2229	2303	2321	2323	2326	2327	2325	2321	2326	2314	2324
	*B* ^1^Σ^+^	2360	2390	2398	2399	2400	2403	2400	2398	2400	2398	2400

a F12-transcorrelated EOM-CCSD approaches with II and VVO PP correlators are contrasted with the standard and conventional F12-correlated EOM-CCSD formalisms.

b Standard EOM-CCSD results from ref. 84.

c EOM-CCSD­(F12) results from ref. 84.

The basis set convergence of equilibrium bond distances (see Table 6) is substantially improved by the addition of explicitly correlated terms; on average the F12 approaches gain the equivalent of a single cardinal number in the orbital basis. The equilibrium bond distances obtained with II and VVO PP variants of the F12 transcorrelation are almost indistinguishable, in contrast to their behavior for correlation energies and the excitation energies discussed earlier. Both transcorrelated approaches produce bond distances that are nearly identical to those from the traditional F12 variant of the EOM-CCSD.

The basis set convergence of harmonic vibrational frequencies is also significantly improved by the F12 explicit correlation; again, on average the F12 approaches gain the equivalent of a single cardinal number in the orbital basis. Unlike the minute differences between the use of II and VVO PP correlators for the equilibrium geometries, there are more noticeable differences between the two correlators, with the PP correlator producing smaller basis set errors. For example, with the daQZ basis the {mean,max} absolute basis set errors obtained with the II and VVO PP correlators are {2.1,6} cm^–1^ and {2.1,5} cm^–1^ respectively. For the majority of states the frequencies obtained with the PP transcorrelator match up well with those from the conventional (F12) variant of EOM-CCSD, but overall the latter is more precise, with the {mean,max} absolute basis set errors of {0.7,3} cm^–1^ with the daQZ basis. This result is not unexpected as the Hättig and Yang implementation is more robust likely due to the more flexible F12 ansatz including many adjustable parameters rather than the fixed amplitude SP ansatz used in the transcorrelated F12 approaches. Note that we recently discovered that the recommended F12 geminal exponents are better suited for the older orbital-invariant CC F12 methods than their SP-ansatz counterparts.^91^ The use of updated geminal exponents with the SP-ansatz-based transcorrelation may help further improve the reported performance relative to that of EOM-CCSD­(F12).

## 5 Summary

The approximate unitary F12-style transcorrelation^36,40^ is a promising approach to address the basis set incompleteness error of correlated electronic states and their properties that produces Hermitian effective Hamiltonians with 2-particle interactions only. The Hermitian and non-Hermitian^48^ F12 transcorrelation frameworks share the core design trait with the traditional F12 models,^13–15,17^ that only the universal (thereby, necessarily, spin-dependent) short-range correlation physics is encoded by a fixed 1-parameter correlator, with the rest of system and state-specific correlation effects modeled by traditional Fock-space expansions.

In this contribution we explored whether it is possible to use F12 transcorrelation for balanced description of ground and low-energy excited states using single-reference coupled-cluster methods. Traditional (nontranscorrelated) explicitly correlated F12 methods capable of treating excited states have been designed before by extending the traditional II ansatz that uses F12 geminals for correlation of pairs of occupied reference orbitals only (thereby undercorrelating the excited states^26^ to include correlation of occupied-virtual reference pairs (IP ansatz).^28–32^ We found that the straightforward extension of the F12 transcorrelation to correlate virtual orbitals results in an unphysical undercorrelation of the ground state, but constraining the approach to use valence-like virtuals only (VVOs) solves this issue and leads to a balanced and accurate description of the ground and low-energy excited states of small systems.

The F12 transcorrelated Hamiltonian based on the VVO PP correlator (in which all pairs involving occupied and valence virtual orbitals are correlated) robustly reduces the basis set errors in correlation energies and in vertical excitation energies (most pronounced for states with Rydberg character). However, for some excited states the residual 1-particle basis set errors can be substantial and will require further refinements of the approach. The properties of individual excited states, such as geometries and especially vibrational frequencies, are also substantially improved by the F12 transcorrelation.

Improvements of the approach focusing on the residual 1-particle basis set incompleteness effects, extensions to special relativity, and assessment of the performance for response properties of molecules will be reported elsewhere.

## Supplementary Material



## Notes

Cartesian geometries of the systems studied in Section 4.1, the equations for transcorrelated Hamiltonian obtained with the IP correlator (eq 10) which have not been reported previously (PDF)
