# Supplementary material for: Toward a Balanced Description of Ground and Excited States with Transcorrelated F12 Methods
Source: J Chem Theory Comput. 2025 Oct 13;21(20):10329–39. doi: 10.1021/acs.jctc.5c01434 (PMC12573748; doi:10.1021/acs.jctc.5c01434)
Supplement: Supplementary file 1 [file ct5c01434_si_001.pdf]

Towards Balanced Description of Ground and Excited States with Transcorrelated F12 Methods

Conner Masteran, Bimal Gaudel, and Edward F. Valeev*

Department of Chemistry, Virginia Tech, Blacksburg, VA 24061

1 Geometries

All Cartesian coordinates are in angstroms.

Table S1: BH

Atom	x	y	z
B	0.00	0.00	0.00
H	0.00	0.00	1.236

Table S2: CH_2O

Atom	x	y	z
C	0.00	0.00	0.00
O	0.00	0.00	1.221104
H	0.00	0.934473	-0.588078
H	0.00	-0.934473	-0.588078

Table S3: N_2

Atom	x	y	z
N	0.00	0.00	0.00
N	0.00	0.00	1.097685

Table S4: CH_2

Atom	x	y	z
C	0.00	0.00	0.00
H	1.1072	0.00	0.00
H	-0.238509836	1.081205299	0.00

Table S5: H_2O

Atom	x	y	z
O	0.00	0.00	0.00
H	0.95623	0.00	0.00
H	-0.478115	0.92380	0.00

2 Equations

The equations for each ansatz are derived using the `SeQuant` symbolic tensor framework and evaluated numerically at run-time. Equations for the II correlator were presented recently in Ref. 1; the PP correlator counterparts can be obtained by trivial modifications. For the IP correlator equations are somewhat different. To validate them we implemented the TC Hamiltonian with IP correlator using two different forms of the correlator:

$$\hat{A}_{\mathrm{IP}} = \frac{1}{2}(G^{i_1 i_2}_{\alpha_1 \alpha_2} \hat{E}^{\alpha_1 \alpha_2}_{i_1 i_2} + 2G^{i_1 e_1}_{\alpha_1 \alpha_2} \hat{E}^{\alpha_1 \alpha_2}_{i_1 e_1} - h.c) = \frac{1}{2}(G^{p'_1 p'_2}_{\alpha_1 \alpha_2} \hat{E}^{\alpha_1 \alpha_2}_{p'_1 p'_2} - 2G^{e_1 p'_1}_{\alpha_2 \alpha_1} \hat{E}^{\alpha_2 \alpha_1}_{e_1 p'_1} - h.c) \tag{S1}$$

The resulting expressions were validated to be equivalent symbolically and numerically.

The evaluation order (factorization into intermediates, etc.) in `SeQuant` is determined by the run-time parameters that control the optimization procedure (such as orbital space sizes). Nevertheless we present the equations of the TC Hamiltonian obtained with the IP correlator in their raw (unfactorized) form for completeness. The equations use the following intermediates that result from the cumulant approximation of the spin-free 3-body replacement:

$$E^{prt}_{qsu} \approx (DE2)^{prt}_{qsu} + (D2E)^{prt}_{qsu} - 2(DDE)^{prt}_{qsu} + C \tag{S2}$$

C contains all the constant terms which cancel out during simplification and will thus be ignored.

$$(DE2)^{prt}_{qsu} = \Gamma^{p}_{q}E^{rt}_{su} + \Gamma^{t}_{u}E^{rp}_{sq} + \Gamma^{r}_{s}E^{pt}_{qu} - \frac{1}{2}\Gamma^{p}_{s}E^{rt}_{qu} - \frac{1}{2}\Gamma^{t}_{q}E^{rp}_{su} - \frac{1}{2}\Gamma^{r}_{q}E^{pt}_{su} - \frac{1}{2}\Gamma^{r}_{u}E^{pt}_{qs} - \frac{1}{2}\Gamma^{p}_{u}E^{tr}_{qs} - \frac{1}{2}\Gamma^{t}_{s}E^{pr}_{qu} \tag{S3}$$

$$(D2E)^{prt}_{qsu} = \Gamma^{rt}_{su}E^{p}_{q} + \Gamma^{rp}_{sq}E^{t}_{u} + \Gamma^{pt}_{qu}E^{r}_{s} - \frac{1}{2}\Gamma^{pt}_{qs}E^{r}_{u} - \frac{1}{2}\Gamma^{rt}_{qu}E^{p}_{s} - \frac{1}{2}\Gamma^{pr}_{qu}E^{t}_{s} - \frac{1}{2}\Gamma^{tr}_{qs}E^{p}_{u} - \frac{1}{2}\Gamma^{pt}_{su}E^{r}_{q} - \frac{1}{2}\Gamma^{rp}_{su}E^{t}_{q} \tag{S4}$$

$$\begin{aligned}(DDE)^{prt}_{qsu} = \\ \Gamma^{t}_{u}\Gamma^{r}_{s}E^{p}_{q} + \Gamma^{p}_{q}\Gamma^{r}_{s}E^{t}_{u} + \Gamma^{t}_{u}\Gamma^{p}_{q}E^{r}_{s} - \\ \frac{1}{2}\Gamma^{p}_{u}\Gamma^{r}_{s}E^{t}_{q} - \frac{1}{2}\Gamma^{p}_{s}\Gamma^{r}_{q}E^{t}_{u} - \frac{1}{2}\Gamma^{p}_{u}\Gamma^{t}_{q}E^{r}_{s} - \frac{1}{2}\Gamma^{r}_{s}\Gamma^{t}_{q}E^{p}_{u} - \frac{1}{2}\Gamma^{t}_{u}\Gamma^{r}_{q}E^{p}_{s} - \frac{1}{2}\Gamma^{t}_{s}\Gamma^{p}_{q}E^{r}_{u} - \frac{1}{2}\Gamma^{t}_{s}\Gamma^{r}_{u}E^{p}_{q} - \frac{1}{2}\Gamma^{p}_{s}\Gamma^{t}_{u}E^{r}_{q} - \frac{1}{2}\Gamma^{r}_{u}\Gamma^{p}_{q}E^{t}_{s} + \\ \frac{1}{4}\Gamma^{r}_{q}\Gamma^{p}_{u}E^{t}_{s} + \frac{1}{4}\Gamma^{r}_{u}\Gamma^{t}_{q}E^{p}_{s} + \frac{1}{4}\Gamma^{p}_{s}\Gamma^{t}_{q}E^{r}_{u} + \frac{1}{4}\Gamma^{p}_{u}\Gamma^{t}_{s}E^{r}_{q} + \frac{1}{4}\Gamma^{t}_{s}\Gamma^{r}_{q}E^{p}_{u} + \frac{1}{4}\Gamma^{r}_{u}\Gamma^{p}_{s}E^{t}_{q}\end{aligned} \tag{S5}$$

All indices are general and Γ refers to the Reduced Density Matrix. Even with these simplifications, the final equations are still quite long. Nonetheless, they are presented here in this compressed form, separated by one and two body pieces.

2.1 1-body TC Hamiltonian with IP correlator

The total number of terms = 34.

$$(E^{e_2 i_1}_{e_1 p_1} G^{x_1 e_1}_{i_1 e_2} h^{p_1}_{x_1} + E^{i_1 i_2}_{e_1 p_1} G^{e_1 x_1}_{i_1 i_2} h^{p_1}_{x_1} \tag{S6}$$

$$+ (D2E)^{i_1 i_2 p_3}_{e_1 p_1 p_2} G^{e_1 x_1}_{i_1 i_2} g^{p_2 p_1}_{p_3 x_1} + (DE2)^{i_1 i_2 p_3}_{e_1 p_1 p_2} G^{e_1 x_1}_{i_1 i_2} g^{p_2 p_1}_{p_3 x_1} \tag{S7}$$

$$+ (D2E)^{i_1 e_2 p_3}_{e_1 p_1 p_2} G^{e_1 x_1}_{i_1 e_2} g^{p_2 p_1}_{p_3 x_1} - 2(DDE)^{e_2 i_1 p_3}_{e_1 p_1 p_2} G^{x_1 e_1}_{i_1 e_2} g^{p_2 p_1}_{p_3 x_1} \tag{S8}$$

$$+ \frac{1}{2} E^{i_1 i_2}_{p_1 p_2} G^{x_1 x_2}_{i_1 i_2} g^{p_1 p_2}_{x_1 x_2} + E^{p_1 e_2}_{i_1 e_1} G^{e_1 i_1}_{e_2 x_1} h^{x_1}_{p_1} \tag{S9}$$

$$- 2(DDE)^{p_2 e_2 p_3}_{i_1 e_1 p_1} G^{e_1 i_1}_{e_2 x_1} g^{p_1 x_1}_{p_3 p_2} + E^{p_1 p_2}_{i_1 e_1} G^{i_1 e_1}_{e_2 x_1} g^{e_2 x_1}_{p_1 p_2} \tag{S10}$$

$$+ E^{e_2 p_1}_{i_1 e_1} G^{i_1 e_1}_{e_2 x_1} h^{x_1}_{p_1} + E^{i_1 e_2}_{e_1 p_1} G^{e_1 x_1}_{i_1 e_2} h^{p_1}_{x_1} \tag{S11}$$

$$+ (DE2)^{e_2 p_2 p_3}_{i_1 e_1 p_1} G^{i_1 e_1}_{e_2 x_1} g^{p_1 x_1}_{p_3 p_2} + E^{p_1 p_2}_{i_1 i_2} G^{i_2 i_1}_{e_1 x_1} g^{e_1 x_1}_{p_2 p_1} \tag{S12}$$

$$- 2(DDE)^{e_2 p_2 p_3}_{i_1 e_1 p_1} G^{i_1 e_1}_{e_2 x_1} g^{p_1 x_1}_{p_3 p_2} + E^{p_1 p_2}_{i_1 e_1} G^{i_1 e_1}_{x_1 x_2} g^{x_1 x_2}_{p_1 p_2} \tag{S13}$$

$$+ (D2E)^{p_2 e_2 p_3}_{i_1 e_1 p_1} G^{e_1 i_1}_{e_2 x_1} g^{p_1 x_1}_{p_3 p_2} + E^{p_1 p_2}_{i_1 e_1} G^{e_1 i_1}_{e_2 x_1} g^{e_2 x_1}_{p_2 p_1} \tag{S14}$$

$$+ E^{i_1 i_2}_{p_1 p_2} G^{e_1 x_1}_{i_2 i_1} g^{p_2 p_1}_{e_1 x_1} + E^{i_1 e_1}_{p_1 p_2} G^{x_1 e_2}_{i_1 e_1} g^{p_2 p_1}_{e_2 x_1} \tag{S15}$$

$$+ E^{i_1 e_1}_{p_1 p_2} G^{x_1 x_2}_{i_1 e_1} g^{p_1 p_2}_{x_1 x_2} + (D2E)^{e_2 i_1 p_3}_{e_1 p_1 p_2} G^{x_1 e_1}_{i_1 e_2} g^{p_2 p_1}_{p_3 x_1} \tag{S16}$$

$$- 2(DDE)^{e_1 p_2 p_3}_{i_1 i_2 p_1} G^{i_1 i_2}_{e_1 x_1} g^{p_1 x_1}_{p_3 p_2} + (DE2)^{e_1 p_2 p_3}_{i_1 i_2 p_1} G^{i_1 i_2}_{e_1 x_1} g^{p_1 x_1}_{p_3 p_2} \tag{S17}$$

$$- 2(DDE)^{i_1 e_2 p_3}_{e_1 p_1 p_2} G^{e_1 x_1}_{i_1 e_2} g^{p_2 p_1}_{p_3 x_1} + (DE2)^{e_2 i_1 p_3}_{e_1 p_1 p_2} G^{x_1 e_1}_{i_1 e_2} g^{p_2 p_1}_{p_3 x_1} \tag{S18}$$

$$+ (D2E)^{e_2 p_2 p_3}_{i_1 e_1 p_1} G^{i_1 e_1}_{e_2 x_1} g^{p_1 x_1}_{p_3 p_2} + (DE2)^{i_1 e_2 p_3}_{e_1 p_1 p_2} G^{e_1 x_1}_{i_1 e_2} g^{p_2 p_1}_{p_3 x_1} \tag{S19}$$

$$+ \frac{1}{2} E^{p_1 p_2}_{i_1 i_2} G^{i_1 i_2}_{x_1 x_2} g^{x_1 x_2}_{p_1 p_2} + E^{i_1 e_1}_{p_1 p_2} G^{e_2 x_1}_{i_1 e_1} g^{p_1 p_2}_{e_2 x_1} \tag{S20}$$

$$- 2(DDE)^{i_1 i_2 p_3}_{e_1 p_1 p_2} G^{e_1 x_1}_{i_1 i_2} g^{p_2 p_1}_{p_3 x_1} + (D2E)^{e_1 p_2 p_3}_{i_1 i_2 p_1} G^{i_1 i_2}_{e_1 x_1} g^{p_1 x_1}_{p_3 p_2} \tag{S21}$$

$$+ E^{e_1 p_1}_{i_1 i_2} G^{i_1 i_2}_{e_1 x_1} h^{x_1}_{p_1} + (DE2)^{p_2 e_2 p_3}_{i_1 e_1 p_1} G^{e_1 i_1}_{e_2 x_1} g^{p_1 x_1}_{p_3 p_2}) \tag{S22}$$

2.2 2-body TC Hamiltonian with IP correlator

The total number of terms = 342.

$$(E^{i_2e_2}_{i_1e_1}G^{e_3x_1}_{i_2e_2}G^{i_1e_1}_{x_2x_1}f^{x_2}_{e_3} + (DDE)^{i_1e_3e_4}_{e_1e_2p_1}G^{e_1x_1}_{i_1e_3}G^{i_2e_2}_{e_4x_1}f^{p_1}_{i_2} \tag{S23}$$

$$- \frac{1}{2}E^{e_2p_1}_{i_1e_1}G^{e_3x_1}_{i_2e_2}G^{e_1i_1}_{e_3x_1}f^{i_2}_{p_1} + \frac{1}{2}(DE2)^{e_2e_3i_2}_{i_1e_1p_1}G^{x_1x_2}_{i_2e_3}G^{i_1e_1}_{e_2x_2}f^{p_1}_{x_1} \tag{S24}$$

$$+ E^{e_2i_2}_{i_1e_1}G^{x_1x_2}_{i_2e_2}G^{i_1e_1}_{x_3x_1}f^{x_3}_{x_2} - 2(DDE)^{e_3i_2e_4}_{i_1e_1e_2}G^{x_1e_2}_{i_2e_4}G^{i_1e_1}_{e_3x_2}f^{x_2}_{x_1} \tag{S25}$$

$$+ E^{e_2i_2}_{i_1e_1}G^{e_3x_1}_{i_2e_2}G^{e_1i_1}_{e_4x_1}f^{e_4}_{e_3} + (DDE)^{e_3i_2p_1}_{i_1e_1e_2}G^{x_1e_2}_{i_2e_4}G^{i_1e_1}_{e_3x_1}f^{e_4}_{p_1} \tag{S26}$$

$$- \frac{1}{2}(D2E)^{e_2p_1i_3}_{i_1i_2e_1}G^{e_1x_1}_{i_3i_4}G^{i_1i_2}_{e_2x_1}f^{i_4}_{p_1} - \frac{1}{2}E^{i_3p_1}_{i_1i_2}G^{e_1x_1}_{i_4i_3}G^{i_2i_1}_{e_1x_1}f^{i_4}_{p_1} \tag{S27}$$

$$+ \frac{1}{2}(D2E)^{i_3p_1e_2}_{i_1i_2e_1}G^{x_1e_1}_{i_3e_2}G^{i_2i_1}_{e_3x_1}f^{e_3}_{p_1} - \frac{1}{2}(D2E)^{i_1e_3e_4}_{e_1e_2p_1}G^{x_1e_2}_{i_1e_3}G^{i_2e_1}_{e_4x_1}f^{p_1}_{i_2} \tag{S28}$$

$$+ (DDE)^{i_2e_3p_1}_{i_1e_1e_2}G^{x_1e_2}_{i_2e_4}G^{e_1i_1}_{e_3x_1}f^{e_4}_{p_1} + (DDE)^{e_2e_3i_2}_{i_1e_1p_1}G^{x_1e_1}_{i_2e_3}G^{i_1e_4}_{e_2x_1}f^{p_1}_{e_4} \tag{S29}$$

$$- \frac{1}{2}E^{i_2e_1}_{i_1p_1}G^{e_2x_1}_{i_2e_1}G^{i_1e_3}_{e_2x_1}f^{p_1}_{e_3} + (DDE)^{e_3i_2p_1}_{i_1e_1e_2}G^{e_1x_1}_{i_2i_3}G^{i_1e_2}_{e_3x_1}f^{i_3}_{p_1} \tag{S30}$$

$$+ E^{i_3i_4}_{i_1i_2}G^{e_1x_1}_{i_4i_3}G^{i_2i_1}_{e_2x_1}f^{e_2}_{e_1} + E^{i_3i_4}_{i_1i_2}G^{x_1x_2}_{i_3i_4}G^{i_1i_2}_{e_1x_2}f^{e_1}_{x_1} \tag{S31}$$

$$- (DDE)^{p_1i_2i_3}_{i_1e_1e_2}G^{e_1x_1}_{i_2i_3}G^{i_1e_2}_{x_2x_1}f^{x_2}_{p_1} + \frac{1}{2}(DE2)^{e_2i_2e_3}_{i_1e_1p_1}G^{x_1x_2}_{i_2e_3}G^{i_1e_1}_{e_2x_1}f^{p_1}_{x_2} \tag{S32}$$

$$+ \frac{1}{2}(DE2)^{p_1i_2e_3}_{i_1e_1e_2}G^{e_1x_1}_{i_2e_3}G^{i_1e_2}_{e_4x_1}f^{e_4}_{p_1} - (DDE)^{e_2e_3i_2}_{i_1e_1p_1}G^{e_4x_1}_{i_2e_3}G^{i_1e_1}_{e_2x_1}f^{p_1}_{e_4} \tag{S33}$$

$$- \frac{1}{2}(D2E)^{e_3e_4i_1}_{e_1e_2p_1}G^{x_1e_1}_{i_1e_3}G^{e_2i_2}_{e_4x_1}f^{p_1}_{i_2} - \frac{1}{2}E^{e_2i_1}_{e_1p_1}G^{x_1x_2}_{i_1e_2}G^{i_2e_1}_{x_1x_2}f^{p_1}_{i_2} \tag{S34}$$

$$+ (DDE)^{e_3i_2p_1}_{i_1e_1e_2}G^{e_2x_1}_{i_3i_2}G^{i_1e_1}_{e_3x_1}f^{i_3}_{p_1} + \frac{1}{2}(DE2)^{p_1i_2i_3}_{i_1e_1e_2}G^{e_1x_1}_{i_2i_3}G^{i_1e_2}_{x_2x_1}f^{x_2}_{p_1} \tag{S35}$$

$$+ \frac{1}{2}(D2E)^{e_3i_2p_1}_{i_1e_1e_2}G^{e_1x_1}_{i_2e_3}G^{e_2i_1}_{e_4x_1}f^{e_4}_{p_1} - \frac{1}{2}(DE2)^{p_1e_3e_4}_{i_1e_1e_2}G^{x_1e_1}_{i_2e_3}G^{e_2i_1}_{e_4x_1}f^{i_2}_{p_1} \tag{S36}$$

$$- (DDE)^{i_3p_1i_4}_{i_1i_2e_1}G^{e_1x_1}_{i_4i_3}G^{i_1i_2}_{x_1x_2}f^{x_2}_{p_1} - \frac{1}{2}E^{i_1e_2}_{e_1p_1}G^{x_1x_2}_{i_1e_2}G^{i_2e_1}_{x_2x_1}f^{p_1}_{i_2} \tag{S37}$$

$$- \frac{1}{2}E^{i_2e_1}_{i_1p_1}G^{x_1e_2}_{i_2e_1}G^{e_3i_1}_{e_2x_1}f^{p_1}_{e_3} - (DDE)^{e_3i_2p_1}_{i_1e_1e_2}G^{e_1x_1}_{i_2e_3}G^{e_2i_1}_{e_4x_1}f^{e_4}_{p_1} \tag{S38}$$

$$- (DDE)^{i_2e_3p_1}_{i_1e_1e_2}G^{x_1e_1}_{i_2e_3}G^{i_1e_2}_{x_1x_2}f^{x_2}_{p_1} + \frac{1}{2}(D2E)^{i_2e_3p_1}_{i_1e_1e_2}G^{x_1e_1}_{i_2e_3}G^{i_1e_2}_{x_1x_2}f^{x_2}_{p_1} \tag{S39}$$

$$+ (DDE)^{p_1i_2e_3}_{i_1e_1e_2}G^{e_1x_1}_{i_2i_3}G^{e_2i_1}_{e_3x_1}f^{i_3}_{p_1} - \frac{1}{2}E^{i_3p_1}_{i_1i_2}G^{x_1x_2}_{i_3i_4}G^{i_1i_2}_{x_1x_2}f^{i_4}_{p_1} \tag{S40}$$

$$- (DDE)^{e_2i_2e_3}_{i_1e_1p_1}G^{x_1x_2}_{i_2e_3}G^{i_1e_1}_{e_2x_1}f^{p_1}_{x_2} + E^{e_2i_2}_{i_1e_1}G^{x_1e_3}_{i_2e_2}G^{i_1e_1}_{e_4x_1}f^{e_4}_{e_3} \tag{S41}$$

$$- \frac{1}{2}E^{i_2e_1}_{i_1p_1}G^{x_1e_2}_{i_2e_1}G^{i_3i_1}_{e_2x_1}f^{p_1}_{i_3} - \frac{1}{2}E^{i_3p_1}_{i_1i_2}G^{e_2x_1}_{i_3e_1}G^{i_1i_2}_{e_2x_1}f^{e_1}_{p_1} \tag{S42}$$

$$+ (D2E)^{i_2i_3e_3}_{i_1e_1e_2}G^{e_1x_1}_{i_3i_2}G^{e_2i_1}_{e_3x_2}f^{x_2}_{x_1} \tag{S43}$$

$$+ (D2E)^{e_3 i_2 e_4}_{i_1 e_1 e_2} G^{e_1 x_1}_{i_2 e_3} G^{e_2 i_1}_{e_4 x_2} f^{x_2}_{x_1} \tag{S44}$$

$$- 2(DDE)^{e_2 e_3 i_3}_{i_1 i_2 e_1} G^{e_1 x_1}_{i_3 e_3} G^{i_1 i_2}_{e_2 x_2} f^{x_2}_{x_1} - \frac{1}{2} E^{i_1 e_2}_{e_1 p_1} G^{x_1 e_3}_{i_1 e_2} G^{i_2 e_1}_{e_3 x_1} f^{p_1}_{i_2} \tag{S45}$$

$$+ (D2E)^{i_3 e_2 i_4}_{i_1 i_2 e_1} G^{e_1 x_1}_{i_4 i_3} G^{i_2 i_1}_{e_2 x_2} f^{x_2}_{x_1} - \frac{1}{2}(D2E)^{e_2 p_1 e_3}_{i_1 i_2 e_1} G^{x_1 e_1}_{i_3 e_3} G^{i_1 i_2}_{e_2 x_1} f^{i_3}_{p_1} \tag{S46}$$

$$+ E^{i_3 i_4}_{i_1 i_2} G^{x_1 x_2}_{i_3 i_4} G^{i_1 i_2}_{x_1 x_3} f^{x_3}_{x_2} + \frac{1}{2}(DE2)^{e_3 i_2 p_1}_{i_1 e_1 e_2} G^{e_1 x_1}_{i_2 e_3} G^{i_1 e_2}_{x_1 x_2} f^{x_2}_{p_1} \tag{S47}$$

$$- \frac{1}{2}(D2E)^{p_1 i_2 e_3}_{i_1 e_1 e_2} G^{e_1 x_1}_{i_2 i_3} G^{e_2 i_1}_{e_3 x_1} f^{i_3}_{p_1} + (D2E)^{e_3 i_2 i_3}_{i_1 e_1 e_2} G^{e_1 x_1}_{i_2 i_3} G^{i_1 e_2}_{e_3 x_2} f^{x_2}_{x_1} \tag{S48}$$

$$- \frac{1}{2}(DE2)^{e_2 p_1 i_3}_{i_1 i_2 e_1} G^{e_1 x_1}_{i_3 e_3} G^{i_1 i_2}_{e_2 x_1} f^{e_3}_{p_1} + \frac{1}{2}(DE2)^{e_2 e_3 i_2}_{i_1 e_1 p_1} G^{e_4 x_1}_{i_2 e_3} G^{i_1 e_1}_{e_2 x_1} f^{p_1}_{e_4} \tag{S49}$$

$$+ (DDE)^{e_3 e_4 i_1}_{e_1 e_2 p_1} G^{x_1 e_1}_{i_1 e_3} G^{e_2 i_2}_{e_4 x_1} f^{p_1}_{i_2} - \frac{1}{2} E^{e_1 i_2}_{i_1 p_1} G^{e_2 x_1}_{i_2 e_1} G^{e_3 i_1}_{e_2 x_1} f^{p_1}_{e_3} \tag{S50}$$

$$- \frac{1}{2}(D2E)^{p_1 e_3 e_4}_{i_1 e_1 e_2} G^{x_1 e_1}_{i_2 e_3} G^{e_2 i_1}_{e_4 x_1} f^{i_2}_{p_1} - \frac{1}{2}(DE2)^{i_2 e_2 e_3}_{i_1 e_1 p_1} G^{x_1 e_1}_{i_2 e_2} G^{i_3 i_1}_{e_3 x_1} f^{p_1}_{i_3} \tag{S51}$$

$$- (DDE)^{i_2 e_2 i_3}_{i_1 e_1 p_1} G^{x_1 x_2}_{i_2 i_3} G^{e_1 i_1}_{e_2 x_1} f^{p_1}_{x_2} - \frac{1}{2}(D2E)^{i_1 i_2 e_3}_{e_1 e_2 p_1} G^{e_1 x_1}_{i_1 i_2} G^{i_3 e_2}_{e_3 x_1} f^{p_1}_{i_3} \tag{S52}$$

$$+ (DDE)^{e_2 p_1 e_3}_{i_1 i_2 e_1} G^{x_1 e_1}_{i_3 e_3} G^{i_1 i_2}_{e_2 x_1} f^{i_3}_{p_1} - \frac{1}{2} E^{i_1 e_2}_{e_1 p_1} G^{e_3 x_1}_{i_1 e_2} G^{e_1 i_2}_{e_3 x_1} f^{p_1}_{i_2} \tag{S53}$$

$$+ E^{i_3 e_1}_{i_1 i_2} G^{x_1 e_2}_{i_3 e_1} G^{i_2 i_1}_{e_3 x_1} f^{e_3}_{e_2} - \frac{1}{2} E^{e_1 p_1}_{i_1 i_2} G^{e_2 x_1}_{i_3 e_1} G^{i_2 i_1}_{e_2 x_1} f^{i_3}_{p_1} \tag{S54}$$

$$+ \frac{1}{2}(DE2)^{p_1 i_2 i_3}_{i_1 e_1 e_2} G^{e_1 x_1}_{i_2 i_3} G^{i_1 e_2}_{e_3 x_1} f^{e_3}_{p_1} + (D2E)^{i_3 e_2 e_3}_{i_1 i_2 e_1} G^{x_1 e_1}_{i_3 e_3} G^{i_2 i_1}_{e_2 x_2} f^{x_2}_{x_1} \tag{S55}$$

$$- \frac{1}{2}(DE2)^{i_3 e_2 p_1}_{i_1 i_2 e_1} G^{e_1 x_1}_{i_4 i_3} G^{i_2 i_1}_{e_2 x_1} f^{i_4}_{p_1} + E^{e_2 i_2}_{i_1 e_1} G^{x_1 x_2}_{i_2 e_2} G^{i_1 e_1}_{e_3 x_1} f^{e_3}_{x_2} \tag{S56}$$

$$- 2(DDE)^{e_3 i_2 i_3}_{i_1 e_1 e_2} G^{e_1 x_1}_{i_2 i_3} G^{i_1 e_2}_{e_3 x_2} f^{x_2}_{x_1} + (DE2)^{e_2 e_3 i_3}_{i_1 i_2 e_1} G^{e_1 x_1}_{i_3 e_3} G^{i_1 i_2}_{e_2 x_2} f^{x_2}_{x_1} \tag{S57}$$

$$- (DDE)^{i_3 e_1 e_2}_{i_1 i_2 p_1} G^{x_1 x_2}_{i_3 e_2} G^{i_2 i_1}_{e_1 x_1} f^{p_1}_{x_2} + E^{i_2 i_3}_{i_1 e_1} G^{e_2 x_1}_{i_2 i_3} G^{i_1 e_1}_{x_2 x_1} f^{x_2}_{e_2} \tag{S58}$$

$$- \frac{1}{2} E^{e_1 i_2}_{i_1 p_1} G^{x_1 e_2}_{i_2 e_1} G^{i_1 i_3}_{e_2 x_1} f^{p_1}_{i_3} - \frac{1}{2}(DE2)^{e_3 i_2 p_1}_{i_1 e_1 e_2} G^{e_1 x_1}_{i_2 i_3} G^{i_1 e_2}_{e_3 x_1} f^{i_3}_{p_1} \tag{S59}$$

$$+ \frac{1}{2}(D2E)^{i_2 i_3 p_1}_{i_1 e_1 e_2} G^{e_1 x_1}_{i_3 i_2} G^{e_2 i_1}_{e_3 x_1} f^{e_3}_{p_1} - \frac{1}{2}(DE2)^{i_1 i_2 e_3}_{e_1 e_2 p_1} G^{e_1 x_1}_{i_1 i_2} G^{i_3 e_2}_{e_3 x_1} f^{p_1}_{i_3} \tag{S60}$$

$$- \frac{1}{2}(D2E)^{i_2 e_2 e_3}_{i_1 e_1 p_1} G^{x_1 e_1}_{i_2 e_2} G^{i_3 i_1}_{e_3 x_1} f^{p_1}_{i_3} + \frac{1}{2}(DE2)^{i_2 e_2 e_3}_{i_1 e_1 p_1} G^{x_1 e_4}_{i_2 e_3} G^{e_1 i_1}_{e_2 x_1} f^{p_1}_{e_4} \tag{S61}$$

$$+ (D2E)^{i_2 e_3 e_4}_{i_1 e_1 e_2} G^{x_1 e_1}_{i_2 e_3} G^{e_2 i_1}_{e_4 x_2} f^{x_2}_{x_1} + \frac{1}{2}(DE2)^{i_2 e_3 p_1}_{i_1 e_1 e_2} G^{x_1 e_1}_{i_2 e_3} G^{i_1 e_2}_{x_1 x_2} f^{x_2}_{p_1} \tag{S62}$$

$$- \frac{1}{2} E^{i_3 p_1}_{i_1 i_2} G^{e_1 x_1}_{i_3 i_4} G^{i_1 i_2}_{e_1 x_1} f^{i_4}_{p_1} + E^{i_2 i_3}_{i_1 e_1} G^{e_2 x_1}_{i_2 i_3} G^{i_1 e_1}_{e_2 x_2} f^{x_2}_{x_1} \tag{S63}$$

$$+ (DE2)^{e_3 i_2 e_4}_{i_1 e_1 e_2} G^{e_1 x_1}_{i_2 e_3} G^{e_2 i_1}_{e_4 x_2} f^{x_2}_{x_1} \tag{S64}$$

$$+ (DDE)^{e_2 i_2 e_3}_{i_1 e_1 p_1} G^{e_1 x_1}_{i_2 e_3} G^{i_1 i_3}_{e_2 x_1} f^{p_1}_{i_3} \tag{S65}$$

$$+ \frac{1}{2}(D2E)^{i_2 e_3 p_1}_{i_1 e_1 e_2} G^{x_1 e_1}_{i_2 e_3} G^{e_2 i_1}_{e_4 x_1} f^{e_4}_{p_1} - 2(DDE)^{i_3 e_2 e_3}_{i_1 i_2 e_1} G^{x_1 e_1}_{i_3 e_3} G^{i_2 i_1}_{e_2 x_2} f^{x_2}_{x_1} \tag{S66}$$

$$+ \frac{1}{2}(DE2)^{e_2 i_2 i_3}_{i_1 e_1 p_1} G^{e_3 x_1}_{i_3 i_2} G^{i_1 e_1}_{e_2 x_1} f^{p_1}_{e_3} - \frac{1}{2} E^{e_2 i_1}_{e_1 p_1} G^{x_1 e_3}_{i_1 e_2} G^{e_1 i_2}_{e_3 x_1} f^{p_1}_{i_2} \tag{S67}$$

$$- \frac{1}{2} E^{e_1 p_1}_{i_1 i_2} G^{x_1 e_2}_{i_3 e_1} G^{i_1 i_2}_{e_2 x_1} f^{i_3}_{p_1} - \frac{1}{2}(DE2)^{i_2 e_3 p_1}_{i_1 e_1 e_2} G^{e_2 x_1}_{i_3 i_2} G^{e_1 i_1}_{e_3 x_1} f^{i_3}_{p_1} \tag{S68}$$

$$+ (DDE)^{e_2 i_2 e_3}_{i_1 e_1 p_1} G^{e_1 x_1}_{i_2 e_2} G^{e_4 i_1}_{e_3 x_1} f^{p_1}_{e_4} + E^{i_2 e_2}_{i_1 e_1} G^{x_1 x_2}_{i_2 e_2} G^{i_1 e_1}_{e_3 x_2} f^{e_3}_{x_1} \tag{S69}$$

$$- (DDE)^{i_3 e_1 e_2}_{i_1 i_2 p_1} G^{x_1 e_3}_{i_3 e_2} G^{i_2 i_1}_{e_1 x_1} f^{p_1}_{e_3} + E^{i_3 e_1}_{i_1 i_2} G^{x_1 e_2}_{i_3 e_1} G^{i_1 i_2}_{x_1 x_2} f^{x_2}_{e_2} \tag{S70}$$

$$+ E^{i_3 i_4}_{i_1 i_2} G^{e_1 x_1}_{i_3 i_4} G^{i_1 i_2}_{e_1 x_2} f^{x_2}_{x_1} + (DDE)^{e_2 e_3 p_1}_{i_1 i_2 e_1} G^{e_1 x_1}_{i_3 e_3} G^{i_1 i_2}_{e_2 x_1} f^{i_3}_{p_1} \tag{S71}$$

$$- \frac{1}{2}(D2E)^{e_2 i_2 i_3}_{i_1 e_1 p_1} G^{e_1 x_1}_{i_2 i_3} G^{i_1 e_3}_{e_2 x_1} f^{p_1}_{e_3} - \frac{1}{2}(DE2)^{i_1 e_3 e_4}_{e_1 e_2 p_1} G^{e_1 x_1}_{i_1 e_4} G^{e_2 i_2}_{e_3 x_1} f^{p_1}_{i_2} \tag{S72}$$

$$+ (DDE)^{i_1 e_3 i_2}_{e_1 e_2 p_1} G^{e_1 x_1}_{i_1 i_2} G^{e_2 i_3}_{e_3 x_1} f^{p_1}_{i_3} + \frac{1}{2}(D2E)^{e_2 p_1 i_3}_{i_1 i_2 e_1} G^{e_1 x_1}_{i_3 e_2} G^{i_2 i_1}_{e_3 x_1} f^{e_3}_{p_1} \tag{S73}$$

$$+ E^{i_3 i_4}_{i_1 i_2} G^{e_1 x_1}_{i_3 i_4} G^{i_2 i_1}_{x_1 x_2} f^{x_2}_{e_1} + (DDE)^{e_2 p_1 i_3}_{i_1 i_2 e_1} G^{e_1 x_1}_{i_3 i_4} G^{i_1 i_2}_{e_2 x_1} f^{i_4}_{p_1} \tag{S74}$$

$$+ \frac{1}{2}(DE2)^{e_2 e_3 i_2}_{i_1 e_1 p_1} G^{x_1 x_2}_{i_2 e_2} G^{e_1 i_1}_{e_3 x_2} f^{p_1}_{x_1} + \frac{1}{2}(D2E)^{e_3 i_2 p_1}_{i_1 e_1 e_2} G^{e_1 x_1}_{i_2 e_3} G^{i_1 e_2}_{x_1 x_2} f^{x_2}_{p_1} \tag{S75}$$

$$- (DDE)^{i_3 e_1 i_4}_{i_1 i_2 p_1} G^{x_1 x_2}_{i_3 i_4} G^{i_2 i_1}_{e_1 x_1} f^{p_1}_{x_2} - \frac{1}{2}(DE2)^{e_3 e_4 p_1}_{i_1 e_1 e_2} G^{e_2 x_1}_{i_2 e_3} G^{e_1 i_1}_{e_4 x_1} f^{i_2}_{p_1} \tag{S76}$$

$$- \frac{1}{2}(D2E)^{e_2 e_3 i_2}_{i_1 e_1 p_1} G^{x_1 e_1}_{i_2 e_3} G^{i_1 e_4}_{e_2 x_1} f^{p_1}_{e_4} + E^{i_2 e_2}_{i_1 e_1} G^{x_1 e_3}_{i_2 e_2} G^{e_1 i_1}_{e_4 x_1} f^{e_4}_{e_3} \tag{S77}$$

$$- \frac{1}{2}(D2E)^{e_2 i_2 e_3}_{i_1 e_1 p_1} G^{e_1 x_1}_{i_2 e_2} G^{e_4 i_1}_{e_3 x_1} f^{p_1}_{e_4} - \frac{1}{2}(DE2)^{e_3 e_4 i_1}_{e_1 e_2 p_1} G^{x_1 e_1}_{i_1 e_3} G^{e_2 i_2}_{e_4 x_1} f^{p_1}_{i_2} \tag{S78}$$

$$- (DDE)^{i_3 p_1 e_2}_{i_1 i_2 e_1} G^{x_1 e_1}_{i_3 e_2} G^{i_1 i_2}_{x_1 x_2} f^{x_2}_{p_1} + \frac{1}{2}(D2E)^{i_2 i_3 p_1}_{i_1 e_1 e_2} G^{e_1 x_1}_{i_3 i_2} G^{i_1 e_2}_{x_1 x_2} f^{x_2}_{p_1} \tag{S79}$$

$$+ (DE2)^{e_3 i_2 i_3}_{i_1 e_1 e_2} G^{e_1 x_1}_{i_2 i_3} G^{i_1 e_2}_{e_3 x_2} f^{x_2}_{x_1} - \frac{1}{2} E^{i_3 p_1}_{i_1 i_2} G^{x_1 e_2}_{i_3 e_1} G^{i_2 i_1}_{e_2 x_1} f^{e_1}_{p_1} \tag{S80}$$

$$+ E^{i_2 e_2}_{i_1 e_1} G^{x_1 x_2}_{i_2 e_2} G^{i_1 e_1}_{x_1 x_3} f^{x_3}_{x_2} + \frac{1}{2}(DE2)^{p_1 i_2 e_3}_{i_1 e_1 e_2} G^{e_1 x_1}_{i_2 e_3} G^{i_1 e_2}_{x_2 x_1} f^{x_2}_{p_1} \tag{S81}$$

$$- \frac{1}{2} E^{i_2 p_1}_{i_1 e_1} G^{e_3 x_1}_{i_2 e_2} G^{i_1 e_1}_{e_3 x_1} f^{e_2}_{p_1} + \frac{1}{2}(D2E)^{p_1 i_2 e_3}_{i_1 e_1 e_2} G^{e_1 x_1}_{i_2 e_3} G^{i_1 e_2}_{x_2 x_1} f^{x_2}_{p_1} \tag{S82}$$

$$+ (DDE)^{e_3 e_4 p_1}_{i_1 e_1 e_2} G^{x_1 e_1}_{i_2 e_4} G^{i_1 e_2}_{e_3 x_1} f^{i_2}_{p_1} + E^{i_2 i_3}_{i_1 e_1} G^{x_1 x_2}_{i_2 i_3} G^{i_1 e_1}_{x_3 x_2} f^{x_3}_{x_1} \tag{S83}$$

$$+ \frac{1}{2}(DE2)^{e_2 p_1 i_3}_{i_1 i_2 e_1} G^{e_1 x_1}_{i_3 e_2} G^{i_1 i_2}_{x_1 x_2} f^{x_2}_{p_1} - \frac{1}{2}(D2E)^{e_3 i_2 p_1}_{i_1 e_1 e_2} G^{e_1 x_1}_{i_2 e_4} G^{i_1 e_2}_{e_3 x_1} f^{e_4}_{p_1} \tag{S84}$$

$$- \frac{1}{2}(D2E)^{i_1 e_3 e_4}_{e_1 e_2 p_1} G^{e_1 x_1}_{i_1 e_3} G^{i_2 e_2}_{e_4 x_1} f^{p_1}_{i_2} \tag{S85}$$

$$+\frac{1}{2}(D2E)^{i_3p_1i_4}_{i_1i_2e_1}G^{e_1x_1}_{i_4i_3}G^{i_1i_2}_{x_1x_2}f^{x_2}_{p_1} \tag{S86}$$

$$+(DDE)^{i_1e_3e_4}_{e_1e_2p_1}G^{x_1e_2}_{i_1e_3}G^{i_2e_1}_{e_4x_1}f^{p_1}_{i_2}+(D2E)^{e_3i_2e_4}_{i_1e_1e_2}G^{x_1e_2}_{i_2e_4}G^{i_1e_1}_{e_3x_2}f^{x_2}_{x_1} \tag{S87}$$

$$-\frac{1}{2}E^{p_1i_2}_{i_1e_1}G^{e_2x_1}_{i_2i_3}G^{e_1i_1}_{e_2x_1}f^{i_3}_{p_1}-\frac{1}{2}(D2E)^{e_3e_4p_1}_{i_1e_1e_2}G^{e_2x_1}_{i_2e_3}G^{e_1i_1}_{e_4x_1}f^{i_2}_{p_1} \tag{S88}$$

$$-\frac{1}{2}(D2E)^{i_1e_3e_4}_{e_1e_2p_1}G^{e_1x_1}_{i_1e_4}G^{e_2i_2}_{e_3x_1}f^{p_1}_{i_2}-\frac{1}{2}E^{e_1i_2}_{i_1p_1}G^{x_1x_2}_{i_2e_1}G^{i_3i_1}_{x_1x_2}f^{p_1}_{i_3} \tag{S89}$$

$$-\frac{1}{2}E^{i_1i_2}_{e_1p_1}G^{e_2x_1}_{i_2i_1}G^{i_3e_1}_{e_2x_1}f^{p_1}_{i_3}+\frac{1}{2}(DE2)^{e_2p_1i_3}_{i_1i_2e_1}G^{e_1x_1}_{i_3e_2}G^{i_2i_1}_{e_3x_1}f^{e_3}_{p_1} \tag{S90}$$

$$+(D2E)^{e_3i_2e_4}_{i_1e_1e_2}G^{e_1x_1}_{i_2e_4}G^{i_1e_2}_{e_3x_2}f^{x_2}_{x_1}-\frac{1}{2}E^{i_2i_3}_{i_1p_1}G^{e_1x_1}_{i_2i_3}G^{i_1i_4}_{e_1x_1}f^{p_1}_{i_4} \tag{S91}$$

$$+(DDE)^{i_3e_2p_1}_{i_1i_2e_1}G^{e_1x_1}_{i_4i_3}G^{i_2i_1}_{e_2x_1}f^{i_4}_{p_1}+\frac{1}{2}(DE2)^{e_2i_2e_3}_{i_1e_1p_1}G^{x_1e_4}_{i_2e_3}G^{i_1e_1}_{e_2x_1}f^{p_1}_{e_4} \tag{S92}$$

$$-(DDE)^{p_1i_2e_3}_{i_1e_1e_2}G^{x_1e_2}_{i_2e_3}G^{i_1e_1}_{x_2x_1}f^{x_2}_{p_1}-\frac{1}{2}E^{i_2p_1}_{i_1e_1}G^{x_1x_2}_{i_2e_2}G^{i_1e_1}_{x_1x_2}f^{e_2}_{p_1} \tag{S93}$$

$$-\frac{1}{2}(DE2)^{e_3i_2p_1}_{i_1e_1e_2}G^{e_1x_1}_{i_2e_4}G^{i_1e_2}_{e_3x_1}f^{e_4}_{p_1}-\frac{1}{2}E^{i_2i_3}_{i_1p_1}G^{x_1x_2}_{i_2i_3}G^{i_1e_1}_{x_1x_2}f^{p_1}_{e_1} \tag{S94}$$

$$+\frac{1}{2}(D2E)^{e_2i_2e_3}_{i_1e_1p_1}G^{x_1x_2}_{i_2e_3}G^{i_1e_1}_{e_2x_1}f^{p_1}_{x_2}+(DDE)^{i_2i_3e_2}_{i_1e_1p_1}G^{e_1x_1}_{i_3i_2}G^{i_4i_1}_{e_2x_1}f^{p_1}_{i_4} \tag{S95}$$

$$+E^{i_2i_3}_{i_1e_1}G^{e_2x_1}_{i_3i_2}G^{e_1i_1}_{e_3x_1}f^{e_3}_{e_2}-\frac{1}{2}E^{p_1i_2}_{i_1e_1}G^{e_2x_1}_{i_3i_2}G^{i_1e_1}_{e_2x_1}f^{i_3}_{p_1} \tag{S96}$$

$$-\frac{1}{2}E^{p_1e_2}_{i_1e_1}G^{x_1x_2}_{i_2e_2}G^{i_1e_1}_{x_1x_2}f^{i_2}_{p_1}-\frac{1}{2}(DE2)^{i_2i_3e_2}_{i_1e_1p_1}G^{e_1x_1}_{i_3i_2}G^{e_3i_1}_{e_2x_1}f^{p_1}_{e_3} \tag{S97}$$

$$+\frac{1}{2}(D2E)^{i_3p_1e_2}_{i_1i_2e_1}G^{x_1e_1}_{i_3e_2}G^{i_1i_2}_{x_1x_2}f^{x_2}_{p_1}+E^{e_2i_2}_{i_1e_1}G^{x_1e_3}_{i_2e_2}G^{i_1e_1}_{x_2x_1}f^{x_2}_{e_3} \tag{S98}$$

$$+E^{i_3e_1}_{i_1i_2}G^{x_1x_2}_{i_3e_1}G^{i_2i_1}_{e_2x_1}f^{e_2}_{x_2}-\frac{1}{2}(DE2)^{e_2e_3i_2}_{i_1e_1p_1}G^{x_1e_1}_{i_2e_3}G^{i_1e_4}_{e_2x_1}f^{p_1}_{e_4} \tag{S99}$$

$$+\frac{1}{2}(DE2)^{e_3i_2p_1}_{i_1e_1e_2}G^{e_1x_1}_{i_2e_3}G^{e_2i_1}_{e_4x_1}f^{e_4}_{p_1}-\frac{1}{2}(D2E)^{e_3i_2p_1}_{i_1e_1e_2}G^{x_1e_2}_{i_2e_4}G^{i_1e_1}_{e_3x_1}f^{e_4}_{p_1} \tag{S100}$$

$$+\frac{1}{2}(D2E)^{i_3e_1e_2}_{i_1i_2p_1}G^{x_1x_2}_{i_3e_2}G^{i_2i_1}_{e_1x_1}f^{p_1}_{x_2}+E^{i_2e_2}_{i_1e_1}G^{x_1e_3}_{i_2e_2}G^{e_1i_1}_{e_3x_2}f^{x_2}_{x_1} \tag{S101}$$

$$-\frac{1}{2}(D2E)^{e_2i_2i_3}_{i_1e_1p_1}G^{e_1x_1}_{i_2i_3}G^{i_1i_4}_{e_2x_1}f^{p_1}_{i_4}-(DDE)^{i_3e_1i_4}_{i_1i_2p_1}G^{e_2x_1}_{i_4i_3}G^{i_2i_1}_{e_1x_1}f^{p_1}_{e_2} \tag{S102}$$

$$-\frac{1}{2}E^{p_1i_2}_{i_1e_1}G^{x_1e_3}_{i_2e_2}G^{i_1e_1}_{e_3x_1}f^{e_2}_{p_1}-(DDE)^{i_3p_1e_2}_{i_1i_2e_1}G^{x_1e_1}_{i_3e_2}G^{i_2i_1}_{e_3x_1}f^{e_3}_{p_1} \tag{S103}$$

$$+(DE2)^{e_3i_2e_4}_{i_1e_1e_2}G^{e_1x_1}_{i_2e_4}G^{i_1e_2}_{e_3x_2}f^{x_2}_{x_1}+\frac{1}{2}(D2E)^{e_2i_2i_3}_{i_1e_1p_1}G^{e_3x_1}_{i_3i_2}G^{i_1e_1}_{e_2x_1}f^{p_1}_{e_3} \tag{S104}$$

$$-\frac{1}{2}E^{i_2e_1}_{i_1p_1}G^{x_1x_2}_{i_2e_1}G^{i_1e_2}_{x_1x_2}f^{p_1}_{e_2}-\frac{1}{2}(DE2)^{e_2p_1e_3}_{i_1i_2e_1}G^{x_1e_1}_{i_3e_3}G^{i_1i_2}_{e_2x_1}f^{i_3}_{p_1} \tag{S105}$$

$$+\frac{1}{2}(D2E)^{i_2e_2e_3}_{i_1e_1p_1}G^{x_1e_4}_{i_2e_3}G^{e_1i_1}_{e_2x_1}f^{p_1}_{e_4} \tag{S106}$$

$$-\frac{1}{2}E^{e_2p_1}_{i_1e_1}G^{x_1x_2}_{i_2e_2}G^{i_1e_1}_{x_2x_1}f^{i_2}_{p_1} \tag{S107}$$

$$-\frac{1}{2}(DE2)^{p_1i_2e_3}_{i_1e_1e_2}G^{e_1x_1}_{i_2i_3}G^{e_2i_1}_{e_3x_1}f^{i_3}_{p_1}-\frac{1}{2}(D2E)^{e_2e_3i_2}_{i_1e_1p_1}G^{x_1e_1}_{i_2e_3}G^{i_1i_3}_{e_2x_1}f^{p_1}_{i_3} \tag{S108}$$

$$+\frac{1}{2}(DE2)^{i_2i_3p_1}_{i_1e_1e_2}G^{e_1x_1}_{i_3i_2}G^{e_2i_1}_{e_3x_1}f^{e_3}_{p_1}+\frac{1}{2}(D2E)^{i_3p_1i_4}_{i_1i_2e_1}G^{e_1x_1}_{i_4i_3}G^{i_2i_1}_{e_2x_1}f^{e_2}_{p_1} \tag{S109}$$

$$-\frac{1}{2}E^{p_1e_2}_{i_1e_1}G^{e_3x_1}_{i_2e_2}G^{i_1e_1}_{e_3x_1}f^{i_2}_{p_1}+E^{e_2i_2}_{i_1e_1}G^{e_3x_1}_{i_2e_2}G^{e_1i_1}_{e_3x_2}f^{x_2}_{x_1} \tag{S110}$$

$$-\frac{1}{2}E^{p_1i_2}_{i_1e_1}G^{x_1x_2}_{i_2e_2}G^{i_1e_1}_{x_2x_1}f^{e_2}_{p_1}+\frac{1}{2}(DE2)^{i_3p_1e_2}_{i_1i_2e_1}G^{x_1e_1}_{i_3e_2}G^{i_2i_1}_{e_3x_1}f^{e_3}_{p_1} \tag{S111}$$

$$-\frac{1}{2}(DE2)^{e_2i_2i_3}_{i_1e_1p_1}G^{e_1x_1}_{i_2i_3}G^{i_1i_4}_{e_2x_1}f^{p_1}_{i_4}+(DDE)^{e_2i_2i_3}_{i_1e_1p_1}G^{e_1x_1}_{i_2i_3}G^{i_1e_3}_{e_2x_1}f^{p_1}_{e_3} \tag{S112}$$

$$+(DDE)^{e_2i_2e_3}_{i_1e_1p_1}G^{e_1x_1}_{i_2e_3}G^{i_1e_4}_{e_2x_1}f^{p_1}_{e_4}+(DDE)^{i_3e_2p_1}_{i_1i_2e_1}G^{x_1e_1}_{i_3e_3}G^{i_2i_1}_{e_2x_1}f^{e_3}_{p_1} \tag{S113}$$

$$+(DDE)^{e_2p_1i_3}_{i_1i_2e_1}G^{e_1x_1}_{i_3e_3}G^{i_1i_2}_{e_2x_1}f^{e_3}_{p_1}+E^{i_3e_1}_{i_1i_2}G^{x_1x_2}_{i_3e_1}G^{i_2i_1}_{x_2x_3}f^{x_3}_{x_1} \tag{S114}$$

$$-\frac{1}{2}E^{i_2p_1}_{i_1e_1}G^{e_2x_1}_{i_3i_2}G^{e_1i_1}_{e_2x_1}f^{i_3}_{p_1}-\frac{1}{2}(DE2)^{e_2i_2e_3}_{i_1e_1p_1}G^{e_1x_1}_{i_2e_3}G^{i_1i_3}_{e_2x_1}f^{p_1}_{i_3} \tag{S115}$$

$$+\frac{1}{2}(D2E)^{i_3e_1i_4}_{i_1i_2p_1}G^{x_1x_2}_{i_3i_4}G^{i_2i_1}_{e_1x_1}f^{p_1}_{x_2}-\frac{1}{2}E^{i_3p_1}_{i_1i_2}G^{x_1x_2}_{i_3e_1}G^{i_1i_2}_{x_1x_2}f^{e_1}_{p_1} \tag{S116}$$

$$+\frac{1}{2}(D2E)^{e_2e_3i_2}_{i_1e_1p_1}G^{e_4x_1}_{i_2e_2}G^{e_1i_1}_{e_3x_1}f^{p_1}_{e_4}-\frac{1}{2}(DE2)^{e_2i_2e_3}_{i_1e_1p_1}G^{e_1x_1}_{i_2e_2}G^{e_4i_1}_{e_3x_1}f^{p_1}_{e_4} \tag{S117}$$

$$-2(DDE)^{e_3i_2e_4}_{i_1e_1e_2}G^{e_1x_1}_{i_2e_3}G^{e_2i_1}_{e_4x_2}f^{x_2}_{x_1}-\frac{1}{2}E^{i_2p_1}_{i_1e_1}G^{e_2x_1}_{i_2i_3}G^{i_1e_1}_{e_2x_1}f^{i_3}_{p_1} \tag{S118}$$

$$-\frac{1}{2}(DE2)^{e_3e_4p_1}_{i_1e_1e_2}G^{x_1e_1}_{i_2e_4}G^{i_1e_2}_{e_3x_1}f^{i_2}_{p_1}+E^{i_3e_1}_{i_1i_2}G^{x_1x_2}_{i_3e_1}G^{i_1i_2}_{x_1x_3}f^{x_3}_{x_2} \tag{S119}$$

$$-\frac{1}{2}E^{e_2p_1}_{i_1e_1}G^{x_1e_3}_{i_2e_2}G^{i_1e_1}_{e_3x_1}f^{i_2}_{p_1}+\frac{1}{2}(D2E)^{i_3e_1i_4}_{i_1i_2p_1}G^{e_2x_1}_{i_4i_3}G^{i_2i_1}_{e_1x_1}f^{p_1}_{e_2} \tag{S120}$$

$$-\frac{1}{2}(DE2)^{e_2i_2i_3}_{i_1e_1p_1}G^{e_1x_1}_{i_2i_3}G^{i_1e_3}_{e_2x_1}f^{p_1}_{e_3}-(DDE)^{i_2e_2e_3}_{i_1e_1p_1}G^{x_1x_2}_{i_2e_3}G^{e_1i_1}_{e_2x_1}f^{p_1}_{x_2} \tag{S121}$$

$$+E^{i_2e_2}_{i_1e_1}G^{x_1x_2}_{i_2e_2}G^{e_1i_1}_{e_3x_1}f^{e_3}_{x_2}-\frac{1}{2}(D2E)^{e_3i_2p_1}_{i_1e_1e_2}G^{e_1x_1}_{i_2i_3}G^{i_1e_2}_{e_3x_1}f^{i_3}_{p_1} \tag{S122}$$

$$+E^{i_2i_3}_{i_1e_1}G^{x_1x_2}_{i_2i_3}G^{e_1i_1}_{e_2x_1}f^{e_2}_{x_2}+\frac{1}{2}(DE2)^{i_3p_1i_4}_{i_1i_2e_1}G^{e_1x_1}_{i_4i_3}G^{i_1i_2}_{x_1x_2}f^{x_2}_{p_1} \tag{S123}$$

$$+\frac{1}{2}(DE2)^{i_2i_3p_1}_{i_1e_1e_2}G^{e_1x_1}_{i_3i_2}G^{i_1e_2}_{x_1x_2}f^{x_2}_{p_1}+E^{i_2e_2}_{i_1e_1}G^{x_1e_3}_{i_2e_2}G^{i_1e_1}_{x_1x_2}f^{x_2}_{e_3} \tag{S124}$$

$$-\frac{1}{2}E^{i_2e_1}_{i_1p_1}G^{x_1x_2}_{i_2e_1}G^{i_1i_3}_{x_1x_2}f^{p_1}_{i_3}-\frac{1}{2}(D2E)^{i_2i_3e_2}_{i_1e_1p_1}G^{e_1x_1}_{i_3i_2}G^{i_4i_1}_{e_2x_1}f^{p_1}_{i_4} \tag{S125}$$

$$+E^{i_2i_3}_{i_1e_1}G^{e_2x_1}_{i_2i_3}G^{i_1e_1}_{e_3x_1}f^{e_3}_{e_2}-(DDE)^{e_3i_2p_1}_{i_1e_1e_2}G^{e_1x_1}_{i_2e_3}G^{i_1e_2}_{x_1x_2}f^{x_2}_{p_1} \tag{S126}$$

$$-\frac{1}{2}E^{e_2i_1}_{e_1p_1}G^{e_3x_1}_{i_1e_2}G^{i_2e_1}_{e_3x_1}f^{p_1}_{i_2} \tag{S127}$$

$$+ (DDE)^{p_1 i_2 e_3}_{i_1 e_1 e_2} G^{e_1 x_1}_{i_2 e_4} G^{e_2 i_1}_{e_3 x_1} f^{e_4}_{p_1} \tag{S128}$$

$$- \frac{1}{2}(DE2)^{i_1 e_3 i_2}_{e_1 e_2 p_1} G^{e_1 x_1}_{i_1 i_2} G^{e_2 i_3}_{e_3 x_1} f^{p_1}_{i_3} - (DDE)^{i_2 i_3 p_1}_{i_1 e_1 e_2} G^{e_1 x_1}_{i_3 i_2} G^{e_2 i_1}_{e_3 x_1} f^{e_3}_{p_1} \tag{S129}$$

$$- \frac{1}{2} E^{p_1 e_2}_{i_1 e_1} G^{x_1 e_3}_{i_2 e_2} G^{e_1 i_1}_{e_3 x_1} f^{i_2}_{p_1} + \frac{1}{2}(DE2)^{i_3 p_1 i_4}_{i_1 i_2 e_1} G^{e_1 x_1}_{i_4 i_3} G^{i_2 i_1}_{e_2 x_1} f^{e_2}_{p_1} \tag{S130}$$

$$+ \frac{1}{2}(DE2)^{i_3 e_1 e_2}_{i_1 i_2 p_1} G^{x_1 x_2}_{i_3 e_2} G^{i_2 i_1}_{e_1 x_1} f^{p_1}_{x_2} - \frac{1}{2}(DE2)^{e_2 e_3 i_2}_{i_1 e_1 p_1} G^{x_1 e_1}_{i_2 e_3} G^{i_1 i_3}_{e_2 x_1} f^{p_1}_{i_3} \tag{S131}$$

$$- \frac{1}{2} E^{i_2 i_3}_{i_1 p_1} G^{e_1 x_1}_{i_3 i_2} G^{i_4 i_1}_{e_1 x_1} f^{p_1}_{i_4} + E^{i_2 e_2}_{i_1 e_1} G^{e_3 x_1}_{i_2 e_2} G^{i_1 e_1}_{e_4 x_1} f^{e_4}_{e_3} \tag{S132}$$

$$- \frac{1}{2}(D2E)^{i_3 e_2 p_1}_{i_1 i_2 e_1} G^{x_1 e_1}_{i_3 e_3} G^{i_2 i_1}_{e_2 x_1} f^{e_3}_{p_1} + (D2E)^{e_2 e_3 i_3}_{i_1 i_2 e_1} G^{e_1 x_1}_{i_3 e_3} G^{i_1 i_2}_{e_2 x_2} f^{x_2}_{x_1} \tag{S133}$$

$$+ \frac{1}{2}(DE2)^{p_1 i_2 e_3}_{i_1 e_1 e_2} G^{x_1 e_2}_{i_2 e_3} G^{i_1 e_1}_{x_2 x_1} f^{x_2}_{p_1} + \frac{1}{2}(D2E)^{p_1 i_2 i_3}_{i_1 e_1 e_2} G^{e_1 x_1}_{i_2 i_3} G^{i_1 e_2}_{e_3 x_1} f^{e_3}_{p_1} \tag{S134}$$

$$- \frac{1}{2} E^{i_2 p_1}_{i_1 e_1} G^{x_1 e_3}_{i_2 e_2} G^{e_1 i_1}_{e_3 x_1} f^{e_2}_{p_1} - \frac{1}{2}(DE2)^{p_1 i_2 e_3}_{i_1 e_1 e_2} G^{e_1 x_1}_{i_2 e_4} G^{e_2 i_1}_{e_3 x_1} f^{e_4}_{p_1} \tag{S135}$$

$$- \frac{1}{2}(DE2)^{e_2 e_3 p_1}_{i_1 i_2 e_1} G^{e_1 x_1}_{i_3 e_3} G^{i_1 i_2}_{e_2 x_1} f^{i_3}_{p_1} - \frac{1}{2}(D2E)^{e_3 e_4 p_1}_{i_1 e_1 e_2} G^{e_2 x_1}_{i_2 e_4} G^{i_1 e_1}_{e_3 x_1} f^{i_2}_{p_1} \tag{S136}$$

$$- \frac{1}{2}(DE2)^{e_2 i_2 e_3}_{i_1 e_1 p_1} G^{e_1 x_1}_{i_2 e_2} G^{i_3 i_1}_{e_3 x_1} f^{p_1}_{i_3} + E^{e_2 i_2}_{i_1 e_1} G^{e_3 x_1}_{i_2 e_2} G^{i_1 e_1}_{x_1 x_2} f^{x_2}_{e_3} \tag{S137}$$

$$- \frac{1}{2}(DE2)^{i_2 e_3 p_1}_{i_1 e_1 e_2} G^{x_1 e_2}_{i_2 e_4} G^{e_1 i_1}_{e_3 x_1} f^{e_4}_{p_1} + \frac{1}{2}(DE2)^{e_1 e_2 i_3}_{i_1 i_2 p_1} G^{x_1 x_2}_{i_3 e_2} G^{i_1 i_2}_{e_1 x_2} f^{p_1}_{x_1} \tag{S138}$$

$$- \frac{1}{2}(DE2)^{i_1 e_3 e_4}_{e_1 e_2 p_1} G^{e_1 x_1}_{i_1 e_3} G^{i_2 e_2}_{e_4 x_1} f^{p_1}_{i_2} + \frac{1}{2}(D2E)^{e_1 e_2 i_3}_{i_1 i_2 p_1} G^{x_1 x_2}_{i_3 e_2} G^{i_1 i_2}_{e_1 x_2} f^{p_1}_{x_1} \tag{S139}$$

$$- \frac{1}{2} E^{i_2 i_3}_{i_1 p_1} G^{e_1 x_1}_{i_3 i_2} G^{e_2 i_1}_{e_1 x_1} f^{p_1}_{e_2} - 2(DDE)^{i_2 i_3 e_3}_{i_1 e_1 e_2} G^{e_1 x_1}_{i_3 i_2} G^{e_2 i_1}_{e_3 x_2} f^{x_2}_{x_1} \tag{S140}$$

$$+ \frac{1}{2}(DE2)^{i_3 e_1 e_2}_{i_1 i_2 p_1} G^{x_1 e_3}_{i_3 e_2} G^{i_2 i_1}_{e_1 x_1} f^{p_1}_{e_3} + \frac{1}{2}(DE2)^{p_1 i_2 e_3}_{i_1 e_1 e_2} G^{x_1 e_2}_{i_2 e_3} G^{i_1 e_1}_{e_4 x_1} f^{e_4}_{p_1} \tag{S141}$$

$$- (DDE)^{e_2 e_3 i_2}_{i_1 e_1 p_1} G^{x_1 x_2}_{i_2 e_2} G^{e_1 i_1}_{e_3 x_2} f^{p_1}_{x_1} - \frac{1}{2}(D2E)^{e_2 i_2 e_3}_{i_1 e_1 p_1} G^{e_1 x_1}_{i_2 e_3} G^{i_1 e_4}_{e_2 x_1} f^{p_1}_{e_4} \tag{S142}$$

$$- \frac{1}{2} E^{e_1 i_2}_{i_1 p_1} G^{x_1 x_2}_{i_2 e_1} G^{i_1 e_2}_{x_2 x_1} f^{p_1}_{e_2} + \frac{1}{2}(D2E)^{e_2 i_2 i_3}_{i_1 e_1 p_1} G^{x_1 x_2}_{i_2 i_3} G^{i_1 e_1}_{e_2 x_1} f^{p_1}_{x_2} \tag{S143}$$

$$- \frac{1}{2}(D2E)^{e_3 i_2 p_1}_{i_1 e_1 e_2} G^{e_2 x_1}_{i_3 i_2} G^{i_1 e_1}_{e_3 x_1} f^{i_3}_{p_1} + E^{i_2 e_2}_{i_1 e_1} G^{e_3 x_1}_{i_2 e_2} G^{i_1 e_1}_{e_3 x_2} f^{x_2}_{x_1} \tag{S144}$$

$$- \frac{1}{2}(D2E)^{p_1 i_2 e_3}_{i_1 e_1 e_2} G^{e_1 x_1}_{i_2 e_4} G^{e_2 i_1}_{e_3 x_1} f^{e_4}_{p_1} + \frac{1}{2}(D2E)^{e_2 i_2 e_3}_{i_1 e_1 p_1} G^{x_1 e_4}_{i_2 e_3} G^{i_1 e_1}_{e_2 x_1} f^{p_1}_{e_4} \tag{S145}$$

$$+ \frac{1}{2}(DE2)^{e_1 e_2 i_3}_{i_1 i_2 p_1} G^{e_3 x_1}_{i_3 e_2} G^{i_1 i_2}_{e_1 x_1} f^{p_1}_{e_3} - \frac{1}{2}(DE2)^{i_2 e_2 e_3}_{i_1 e_1 p_1} G^{x_1 e_1}_{i_2 e_2} G^{e_4 i_1}_{e_3 x_1} f^{p_1}_{e_4} \tag{S146}$$

$$+ \frac{1}{2}(D2E)^{i_2 e_2 e_3}_{i_1 e_1 p_1} G^{x_1 x_2}_{i_2 e_3} G^{e_1 i_1}_{e_2 x_1} f^{p_1}_{x_2} - \frac{1}{2}(DE2)^{e_2 i_2 e_3}_{i_1 e_1 p_1} G^{e_1 x_1}_{i_2 e_3} G^{i_1 e_4}_{e_2 x_1} f^{p_1}_{e_4} \tag{S147}$$

$$+ \frac{1}{2}(DE2)^{i_2 e_2 i_3}_{i_1 e_1 p_1} G^{x_1 x_2}_{i_2 i_3} G^{e_1 i_1}_{e_2 x_1} f^{p_1}_{x_2} \tag{S148}$$

$$+ (DDE)^{i_2e_3p_1}_{i_1e_1e_2} G^{e_2x_1}_{i_3i_2} G^{e_1i_1}_{e_3x_1} f^{i_3}_{p_1} \tag{S149}$$

$$+ E^{i_3e_1}_{i_1i_2} G^{e_2x_1}_{i_3e_1} G^{i_2i_1}_{x_1x_2} f^{x_2}_{e_2} - (DDE)^{e_2i_2i_3}_{i_1e_1p_1} G^{x_1x_2}_{i_2i_3} G^{i_1e_1}_{e_2x_1} f^{p_1}_{x_2} \tag{S150}$$

$$+ E^{i_2e_2}_{i_1e_1} G^{x_1x_2}_{i_2e_2} G^{i_1e_1}_{x_3x_2} f^{x_3}_{x_1} + \frac{1}{2}(D2E)^{i_2e_2i_3}_{i_1e_1p_1} G^{e_3x_1}_{i_3i_2} G^{e_1i_1}_{e_2x_1} f^{p_1}_{e_3} \tag{S151}$$

$$+ \frac{1}{2}(D2E)^{i_3e_1e_2}_{i_1i_2p_1} G^{x_1e_3}_{i_3e_2} G^{i_2i_1}_{e_1x_1} f^{p_1}_{e_3} + (DDE)^{e_3e_4p_1}_{i_1e_1e_2} G^{e_2x_1}_{i_2e_4} G^{i_1e_1}_{e_3x_1} f^{i_2}_{p_1} \tag{S152}$$

$$- (DDE)^{p_1i_2e_3}_{i_1e_1e_2} G^{e_1x_1}_{i_2e_3} G^{i_1e_2}_{x_2x_1} f^{x_2}_{p_1} - \frac{1}{2}(DE2)^{e_3i_2p_1}_{i_1e_1e_2} G^{e_2x_1}_{i_3i_2} G^{i_1e_1}_{e_3x_1} f^{i_3}_{p_1} \tag{S153}$$

$$- (DDE)^{e_2i_2e_3}_{i_1e_1p_1} G^{x_1e_4}_{i_2e_3} G^{i_1e_1}_{e_2x_1} f^{p_1}_{e_4} - \frac{1}{2}E^{i_2p_1}_{i_1e_1} G^{x_1x_2}_{i_2i_3} G^{i_1e_1}_{x_1x_2} f^{i_3}_{p_1} \tag{S154}$$

$$+ E^{i_2i_3}_{i_1e_1} G^{x_1x_2}_{i_2i_3} G^{i_1e_1}_{e_2x_2} f^{e_2}_{x_1} - 2(DDE)^{i_3e_2i_4}_{i_1i_2e_1} G^{e_1x_1}_{i_4i_3} G^{i_2i_1}_{e_2x_2} f^{x_2}_{x_1} \tag{S155}$$

$$+ \frac{1}{2}(D2E)^{p_1i_2i_3}_{i_1e_1e_2} G^{e_1x_1}_{i_2i_3} G^{i_1e_2}_{x_2x_1} f^{x_2}_{p_1} + \frac{1}{2}(D2E)^{e_1e_2i_3}_{i_1i_2p_1} G^{e_3x_1}_{i_3e_2} G^{i_1i_2}_{e_1x_1} f^{p_1}_{e_3} \tag{S156}$$

$$- (DDE)^{i_2i_3p_1}_{i_1e_1e_2} G^{e_1x_1}_{i_3i_2} G^{i_1e_2}_{x_1x_2} f^{x_2}_{p_1} - \frac{1}{2}E^{i_2i_3}_{i_1p_1} G^{e_1x_1}_{i_2i_3} G^{i_1e_2}_{e_1x_1} f^{p_1}_{e_2} \tag{S157}$$

$$- \frac{1}{2}(D2E)^{e_2i_2e_3}_{i_1e_1p_1} G^{e_1x_1}_{i_2e_2} G^{i_3i_1}_{e_3x_1} f^{p_1}_{i_3} - (DDE)^{i_2e_2e_3}_{i_1e_1p_1} G^{x_1e_4}_{i_2e_3} G^{e_1i_1}_{e_2x_1} f^{p_1}_{e_4} \tag{S158}$$

$$- (DDE)^{p_1i_2i_3}_{i_1e_1e_2} G^{e_1x_1}_{i_2i_3} G^{i_1e_2}_{e_3x_1} f^{e_3}_{p_1} + \frac{1}{2}(DE2)^{i_2e_2i_3}_{i_1e_1p_1} G^{e_3x_1}_{i_3i_2} G^{e_1i_1}_{e_2x_1} f^{p_1}_{e_3} \tag{S159}$$

$$+ (DDE)^{e_2i_2i_3}_{i_1e_1p_1} G^{e_1x_1}_{i_2i_3} G^{i_1i_4}_{e_2x_1} f^{p_1}_{i_4} + \frac{1}{2}(DE2)^{i_2e_3p_1}_{i_1e_1e_2} G^{x_1e_1}_{i_2e_3} G^{e_2i_1}_{e_4x_1} f^{e_4}_{p_1} \tag{S160}$$

$$+ (DDE)^{i_2e_2e_3}_{i_1e_1p_1} G^{x_1e_1}_{i_2e_2} G^{e_4i_1}_{e_3x_1} f^{p_1}_{e_4} - \frac{1}{2}(DE2)^{e_2p_1i_3}_{i_1i_2e_1} G^{e_1x_1}_{i_3i_4} G^{i_1i_2}_{e_2x_1} f^{i_4}_{p_1} \tag{S161}$$

$$+ E^{i_3e_1}_{i_1i_2} G^{x_1e_2}_{i_3e_1} G^{i_2i_1}_{e_2x_2} f^{x_2}_{x_1} + \frac{1}{2}(D2E)^{e_2e_3i_2}_{i_1e_1p_1} G^{x_1x_2}_{i_2e_2} G^{e_1i_1}_{e_3x_2} f^{p_1}_{x_1} \tag{S162}$$

$$+ (DDE)^{e_3e_4p_1}_{i_1e_1e_2} G^{e_2x_1}_{i_2e_3} G^{e_1i_1}_{e_4x_1} f^{i_2}_{p_1} - \frac{1}{2}(DE2)^{i_3e_2p_1}_{i_1i_2e_1} G^{x_1e_1}_{i_3e_3} G^{i_2i_1}_{e_2x_1} f^{e_3}_{p_1} \tag{S163}$$

$$+ \frac{1}{2}(D2E)^{e_2e_3i_2}_{i_1e_1p_1} G^{e_4x_1}_{i_2e_3} G^{i_1e_1}_{e_2x_1} f^{p_1}_{e_4} - (DDE)^{i_3p_1i_4}_{i_1i_2e_1} G^{e_1x_1}_{i_4i_3} G^{i_2i_1}_{e_2x_1} f^{e_2}_{p_1} \tag{S164}$$

$$+ \frac{1}{2}(DE2)^{i_3e_1i_4}_{i_1i_2p_1} G^{x_1x_2}_{i_3i_4} G^{i_2i_1}_{e_1x_1} f^{p_1}_{x_2} - \frac{1}{2}(D2E)^{i_2e_2e_3}_{i_1e_1p_1} G^{x_1e_1}_{i_2e_2} G^{e_4i_1}_{e_3x_1} f^{p_1}_{e_4} \tag{S165}$$

$$- (DDE)^{p_1i_2e_3}_{i_1e_1e_2} G^{e_1x_1}_{i_2e_3} G^{i_1e_2}_{e_4x_1} f^{e_4}_{p_1} - \frac{1}{2}E^{i_1i_2}_{e_1p_1} G^{e_2x_1}_{i_1i_2} G^{e_1i_3}_{e_2x_1} f^{p_1}_{i_3} \tag{S166}$$

$$+ E^{i_2i_3}_{i_1e_1} G^{e_2x_1}_{i_3i_2} G^{e_1i_1}_{e_2x_2} f^{x_2}_{x_1} + (DE2)^{i_3e_2i_4}_{i_1i_2e_1} G^{e_1x_1}_{i_4i_3} G^{i_2i_1}_{e_2x_2} f^{x_2}_{x_1} \tag{S167}$$

$$- (DDE)^{i_2e_2i_3}_{i_1e_1p_1} G^{e_3x_1}_{i_3i_2} G^{e_1i_1}_{e_2x_1} f^{p_1}_{e_3} - \frac{1}{2}(D2E)^{e_2e_3p_1}_{i_1i_2e_1} G^{e_1x_1}_{i_3e_3} G^{i_1i_2}_{e_2x_1} f^{i_3}_{p_1} \tag{S168}$$

$$- (DDE)^{e_2e_3i_2}_{i_1e_1p_1} G^{x_1x_2}_{i_2e_3} G^{i_1e_1}_{e_2x_2} f^{p_1}_{x_1} \tag{S169}$$

$$+\frac{1}{2}(D2E)^{i_2e_2i_3}_{i_1e_1p_1}G^{x_1x_2}_{i_2i_3}G^{e_1i_1}_{e_2x_1}f^{p_1}_{x_2} \tag{S170}$$

$$-\frac{1}{2}(DE2)^{i_1e_3e_4}_{e_1e_2p_1}G^{x_1e_2}_{i_1e_3}G^{i_2e_1}_{e_4x_1}f^{p_1}_{i_2}-(DDE)^{e_2i_2i_3}_{i_1e_1p_1}G^{e_3x_1}_{i_3i_2}G^{i_1e_1}_{e_2x_1}f^{p_1}_{e_3} \tag{S171}$$

$$-\frac{1}{2}(DE2)^{e_3i_2p_1}_{i_1e_1e_2}G^{x_1e_2}_{i_2e_4}G^{i_1e_1}_{e_3x_1}f^{e_4}_{p_1}+(DDE)^{e_2i_2e_3}_{i_1e_1p_1}G^{e_1x_1}_{i_2e_2}G^{i_3i_1}_{e_3x_1}f^{p_1}_{i_3} \tag{S172}$$

$$-\frac{1}{2}(D2E)^{i_1e_3i_2}_{e_1e_2p_1}G^{e_1x_1}_{i_1i_2}G^{e_2i_3}_{e_3x_1}f^{p_1}_{i_3}-(DDE)^{e_1e_2i_3}_{i_1i_2p_1}G^{x_1x_2}_{i_3e_2}G^{i_1i_2}_{e_1x_2}f^{p_1}_{x_1} \tag{S173}$$

$$-\frac{1}{2}E^{p_1i_2}_{i_1e_1}G^{x_1x_2}_{i_2i_3}G^{i_1e_1}_{x_2x_1}f^{i_3}_{p_1}-2(DDE)^{i_2e_3e_4}_{i_1e_1e_2}G^{x_1e_1}_{i_2e_3}G^{e_2i_1}_{e_4x_2}f^{x_2}_{x_1} \tag{S174}$$

$$-\frac{1}{2}(DE2)^{i_2i_3e_2}_{i_1e_1p_1}G^{e_1x_1}_{i_3i_2}G^{i_4i_1}_{e_2x_1}f^{p_1}_{i_4}+E^{i_2i_3}_{i_1e_1}G^{e_2x_1}_{i_3i_2}G^{i_1e_1}_{x_1x_2}f^{x_2}_{e_2} \tag{S175}$$

$$-\frac{1}{2}(D2E)^{e_3e_4p_1}_{i_1e_1e_2}G^{x_1e_1}_{i_2e_4}G^{i_1e_2}_{e_3x_1}f^{i_2}_{p_1}+(DE2)^{i_2i_3e_3}_{i_1e_1e_2}G^{e_1x_1}_{i_3i_2}G^{e_2i_1}_{e_3x_2}f^{x_2}_{x_1} \tag{S176}$$

$$-\frac{1}{2}E^{i_2i_3}_{i_1p_1}G^{x_1x_2}_{i_2i_3}G^{i_1i_4}_{x_1x_2}f^{p_1}_{i_4}-(DDE)^{e_2p_1i_3}_{i_1i_2e_1}G^{e_1x_1}_{i_3e_2}G^{i_2i_1}_{e_3x_1}f^{e_3}_{p_1} \tag{S177}$$

$$+\frac{1}{2}(DE2)^{i_3e_1i_4}_{i_1i_2p_1}G^{e_2x_1}_{i_4i_3}G^{i_2i_1}_{e_1x_1}f^{p_1}_{e_2}+(DDE)^{i_2e_2e_3}_{i_1e_1p_1}G^{x_1e_1}_{i_2e_2}G^{i_3i_1}_{e_3x_1}f^{p_1}_{i_3} \tag{S178}$$

$$-(DDE)^{e_2p_1i_3}_{i_1i_2e_1}G^{e_1x_1}_{i_3e_2}G^{i_1i_2}_{x_1x_2}f^{x_2}_{p_1}+E^{e_2i_2}_{i_1e_1}G^{x_1x_2}_{i_2e_2}G^{i_1e_1}_{x_2x_3}f^{x_3}_{x_1} \tag{S179}$$

$$+(DDE)^{i_1i_2e_3}_{e_1e_2p_1}G^{e_1x_1}_{i_1i_2}G^{i_3e_2}_{e_3x_1}f^{p_1}_{i_3}-\frac{1}{2}(D2E)^{i_3e_2p_1}_{i_1i_2e_1}G^{e_1x_1}_{i_4i_3}G^{i_2i_1}_{e_2x_1}f^{i_4}_{p_1} \tag{S180}$$

$$-(DDE)^{e_1e_2i_3}_{i_1i_2p_1}G^{e_3x_1}_{i_3e_2}G^{i_1i_2}_{e_1x_1}f^{p_1}_{e_3}-(DDE)^{p_1i_2e_3}_{i_1e_1e_2}G^{x_1e_2}_{i_2e_3}G^{i_1e_1}_{e_4x_1}f^{e_4}_{p_1} \tag{S181}$$

$$+(DDE)^{e_3i_2p_1}_{i_1e_1e_2}G^{e_1x_1}_{i_2e_4}G^{i_1e_2}_{e_3x_1}f^{e_4}_{p_1}-\frac{1}{2}E^{e_1i_2}_{i_1p_1}G^{x_1e_2}_{i_2e_1}G^{i_1e_3}_{e_2x_1}f^{p_1}_{e_3} \tag{S182}$$

$$+\frac{1}{2}(D2E)^{p_1i_2e_3}_{i_1e_1e_2}G^{x_1e_2}_{i_2e_3}G^{i_1e_1}_{e_4x_1}f^{e_4}_{p_1}+\frac{1}{2}(D2E)^{p_1i_2e_3}_{i_1e_1e_2}G^{x_1e_2}_{i_2e_3}G^{i_1e_1}_{x_2x_1}f^{x_2}_{p_1} \tag{S183}$$

$$-\frac{1}{2}(D2E)^{e_2p_1i_3}_{i_1i_2e_1}G^{e_1x_1}_{i_3e_3}G^{i_1i_2}_{e_2x_1}f^{e_3}_{p_1}+(DDE)^{i_1e_3e_4}_{e_1e_2p_1}G^{e_1x_1}_{i_1e_4}G^{e_2i_2}_{e_3x_1}f^{p_1}_{i_2} \tag{S184}$$

$$-(DDE)^{i_2e_3p_1}_{i_1e_1e_2}G^{x_1e_1}_{i_2e_3}G^{e_2i_1}_{e_4x_1}f^{e_4}_{p_1}-2(DDE)^{e_3i_2e_4}_{i_1e_1e_2}G^{e_1x_1}_{i_2e_4}G^{i_1e_2}_{e_3x_2}f^{x_2}_{x_1} \tag{S185}$$

$$+\frac{1}{2}(DE2)^{e_2e_3i_2}_{i_1e_1p_1}G^{e_4x_1}_{i_2e_2}G^{e_1i_1}_{e_3x_1}f^{p_1}_{e_4}+(DE2)^{i_3e_2e_3}_{i_1i_2e_1}G^{x_1e_1}_{i_3e_3}G^{i_2i_1}_{e_2x_2}f^{x_2}_{x_1} \tag{S186}$$

$$+\frac{1}{2}(D2E)^{e_2p_1i_3}_{i_1i_2e_1}G^{e_1x_1}_{i_3e_2}G^{i_1i_2}_{x_1x_2}f^{x_2}_{p_1}+(DDE)^{e_2e_3i_2}_{i_1e_1p_1}G^{x_1e_1}_{i_2e_3}G^{i_1i_3}_{e_2x_1}f^{p_1}_{i_3} \tag{S187}$$

$$-\frac{1}{2}(D2E)^{i_2e_3p_1}_{i_1e_1e_2}G^{x_1e_2}_{i_2e_4}G^{e_1i_1}_{e_3x_1}f^{e_4}_{p_1}-\frac{1}{2}E^{e_1i_2}_{i_1p_1}G^{e_2x_1}_{i_2e_1}G^{i_3i_1}_{e_2x_1}f^{p_1}_{i_3} \tag{S188}$$

$$+E^{i_3e_1}_{i_1i_2}G^{e_2x_1}_{i_3e_1}G^{i_1i_2}_{e_3x_1}f^{e_3}_{e_2}-\frac{1}{2}E^{i_1i_2}_{e_1p_1}G^{x_1x_2}_{i_1i_2}G^{i_3e_1}_{x_2x_1}f^{p_1}_{i_3} \tag{S189}$$

$$-\frac{1}{2}(D2E)^{i_2i_3e_2}_{i_1e_1p_1}G^{e_1x_1}_{i_3i_2}G^{e_3i_1}_{e_2x_1}f^{p_1}_{e_3} \tag{S190}$$

$$+ E^{e_2i_2}_{i_1e_1} G^{x_1x_2}_{i_2e_2} G^{e_1i_1}_{e_3x_2} f^{e_3}_{x_1} \tag{S191}$$

$$- \frac{1}{2} E^{e_1p_1}_{i_1i_2} G^{x_1x_2}_{i_3e_1} G^{i_2i_1}_{x_1x_2} f^{i_3}_{p_1} + (DE2)^{e_3i_2e_4}_{i_1e_1e_2} G^{x_1e_2}_{i_2e_4} G^{i_1e_1}_{e_3x_2} f^{x_2}_{x_1} \tag{S192}$$

$$- \frac{1}{2} E^{i_2e_1}_{i_1p_1} G^{e_2x_1}_{i_2e_1} G^{i_1i_3}_{e_2x_1} f^{p_1}_{i_3} + (DDE)^{i_2i_3e_2}_{i_1e_1p_1} G^{e_1x_1}_{i_3i_2} G^{e_3i_1}_{e_2x_1} f^{p_1}_{e_3} \tag{S193}$$

$$- \frac{1}{2} (D2E)^{e_2i_2e_3}_{i_1e_1p_1} G^{e_1x_1}_{i_2e_3} G^{i_1i_3}_{e_2x_1} f^{p_1}_{i_3} + (DE2)^{i_2e_3e_4}_{i_1e_1e_2} G^{x_1e_1}_{i_2e_3} G^{e_2i_1}_{e_4x_2} f^{x_2}_{x_1} \tag{S194}$$

$$+ \frac{1}{2} (DE2)^{i_3p_1e_2}_{i_1i_2e_1} G^{x_1e_1}_{i_3e_2} G^{i_1i_2}_{x_1x_2} f^{x_2}_{p_1} + \frac{1}{2} (DE2)^{e_2i_2i_3}_{i_1e_1p_1} G^{x_1x_2}_{i_2i_3} G^{i_1e_1}_{e_2x_1} f^{p_1}_{x_2} \tag{S195}$$

$$+ (DDE)^{p_1e_3e_4}_{i_1e_1e_2} G^{x_1e_1}_{i_2e_3} G^{e_2i_1}_{e_4x_1} f^{i_2}_{p_1} + E^{i_3e_1}_{i_1i_2} G^{x_1x_2}_{i_3e_1} G^{i_1i_2}_{e_2x_2} f^{e_2}_{x_1} \tag{S196}$$

$$+ \frac{1}{2} (D2E)^{e_2e_3i_2}_{i_1e_1p_1} G^{x_1x_2}_{i_2e_3} G^{i_1e_1}_{e_2x_2} f^{p_1}_{x_1} + E^{e_2i_2}_{i_1e_1} G^{x_1e_3}_{i_2e_2} G^{i_1e_1}_{e_3x_2} f^{x_2}_{x_1} \tag{S197}$$

$$- \frac{1}{2} (D2E)^{i_2e_3p_1}_{i_1e_1e_2} G^{e_2x_1}_{i_3i_2} G^{e_1i_1}_{e_3x_1} f^{i_3}_{p_1} - \frac{1}{2} (DE2)^{e_3e_4p_1}_{i_1e_1e_2} G^{e_2x_1}_{i_2e_4} G^{i_1e_1}_{e_3x_1} f^{i_2}_{p_1} \tag{S198}$$

$$- (DDE)^{e_2e_3i_2}_{i_1e_1p_1} G^{e_4x_1}_{i_2e_2} G^{e_1i_1}_{e_3x_1} f^{p_1}_{e_4} + E^{i_3e_1}_{i_1i_2} G^{e_2x_1}_{i_3e_1} G^{i_1i_2}_{e_2x_2} f^{x_2}_{x_1} \tag{S199}$$

$$- \frac{1}{2} E^{p_1i_2}_{i_1e_1} G^{e_3x_1}_{i_2e_2} G^{e_1i_1}_{e_3x_1} f^{e_2}_{p_1} + \frac{1}{2} (DE2)^{i_2e_2e_3}_{i_1e_1p_1} G^{x_1x_2}_{i_2e_3} G^{e_1i_1}_{e_2x_1} f^{p_1}_{x_2} \tag{S200}$$

$$+ E^{i_2i_3}_{i_1e_1} G^{x_1x_2}_{i_2i_3} G^{i_1e_1}_{x_1x_3} f^{x_3}_{x_2} + \frac{1}{2} (D2E)^{p_1i_2e_3}_{i_1e_1e_2} G^{e_1x_1}_{i_2e_3} G^{i_1e_2}_{e_4x_1} f^{e_4}_{p_1}) \tag{S201}$$

References

(1) Masteran, C.; Kumar, A.; Teke, N.; Gaudel, B.; Yanai, T.; Valeev, E. F. Comment on "Canonical Transcorrelated Theory with Projected Slater-type Geminals" [J. Chem. Phys. 136, 084107 (2012)]. *J. Chem. Phys.* **2023**, *158*, 057101.
